# Projected health effects of realistic dietary changes to address freshwater constraints in India: a modelling study

**DOI:** 10.1016/S2542-5196(17)30001-3

**Published:** 2017-04

**Authors:** James Milner, Edward J M Joy, Rosemary Green, Francesca Harris, Lukasz Aleksandrowicz, Sutapa Agrawal, Pete Smith, Andy Haines, Alan D Dangour

**Affiliations:** a Department of Social and Environmental Health Research, London School of Hygiene & Tropical Medicine, London, UK; b Department of Population Health, London School of Hygiene & Tropical Medicine, London, UK; c Leverhulme Centre for Integrative Research on Agriculture and Health, London, UK; d Public Health Foundation of India, Delhi National Capital Region, Institutional Area Gurgaon, Haryana, India; e Institute of Biological and Environmental Sciences, University of Aberdeen, Aberdeen, UK

## Abstract

**Background:**

The availability of freshwater for irrigation in the Indian agricultural sector is expected to decline over the coming decades. This might have implications for food production in India, with subsequent effects on diets and health. We identify realistic and healthy dietary changes that could enhance the resilience of the Indian food system to future decreases in water availability.

**Methods:**

In this modelling study, we optimised typical dietary patterns in an Indian population sample to meet projected decreases in the availability of water per person for irrigation (blue water footprint) due to population growth (to 2025 and 2050). The optimised diets met nutritional guidelines and minimised deviation from existing patterns. Resulting changes in life-years lost due to coronary heart disease, stroke, diabetes, and cancers were modelled using life tables, and changes in greenhouse gas emissions associated with the production of diets were estimated. The primary outcomes of the model were changes in life-years per 100 000 total population over 40 years (to 2050).

**Findings:**

The optimised diets had up to 30% lower blue water footprints and generally contained lower amounts of wheat, dairy, and poultry, and increased amounts of legumes. In the 2050 scenario, adoption of these diets would on average result in 6800 life-years gained per 100 000 total population (95% CI 1600–13 100) over 40 years. The dietary changes were accompanied by reductions in greenhouse gas emissions. The magnitude of the health and environmental effects varied between dietary patterns.

**Interpretation:**

Modest changes in diets could help to address projected reductions in the availability of freshwater for irrigation in India. These dietary changes could also simultaneously reduce diet-related greenhouse gas emissions and improve diet-related health outcomes.

**Funding:**

Wellcome Trust.

## Introduction

In many parts of the world, groundwater resources are being depleted faster than they can be replenished, with some of the highest rates of depletion in areas of high agricultural productivity.^1^ In India, the proportion of available freshwater used for agricultural production might already be unsustainably high,^2^ and water availability per person is projected to decline substantially over the coming decades.^3^ The potential effects of freshwater shortages on India's ability to produce an adequate quantity and quality of food for its population are significant not least because India already has a large double burden of malnutrition. Although prevalence of undernutrition remains high, problems because of overweight and obesity are increasing, especially in wealthier, urban populations.^4^ India faces a growing challenge to shape a food system that is resilient to the combined effects of dietary and epidemiological transitions, population growth, and environmental change. One potential solution to support India's food system tackle falling groundwater resources might be to modify existing dietary patterns.

A number of previous studies have quantified the health and environmental effects of population-level dietary modifications, commonly with the intention of reducing greenhouse gas emissions.^5^ Most previous work in this area has considered shifts in consumption of broad food groups, such as meat, and recently more advanced methods have been used to optimise dietary patterns to achieve health and greenhouse gas reduction targets.^6^ A broad consensus has emerged, typically from studies in high-income countries, that moderating consumption of animal-source foods and increasing consumption of fruits and vegetables, can reduce greenhouse gas emissions and has the cobenefit of improving population health.

Few studies have attempted to derive optimised diets that reduce water use in their production, and to our knowledge, no assessment of the potential effects of such optimised diets on health has been done.^7^ Here, we investigate the resilience of the Indian food system to future changes in the availability of groundwater for agricultural irrigation. Specifically, we optimise typical dietary patterns in India to limit agricultural groundwater use in line with projected reductions in groundwater availability per person and assess the associated effects on greenhouse gas emissions and diet-related health in adults. We focus on ground and surface freshwater used for irrigation (blue water footprints) because irrigation is crucial to food production in India and the depletion of groundwater in food-producing regions is of concern.^2^, ^3^

**Research in context**

**Evidence before this study**

We conducted informal searches of published literature and reports by relevant organisations and were unable to identify estimates of the environmental impacts of typical Indian dietary patterns. We have estimated and previously reported mixed environmental impacts of Indian diets and agricultural production—per person diet-related greenhouse gas emissions are low compared with western diets, but agriculture-related water use is high. National estimates suggest that per person availability of freshwater for agricultural irrigation is projected to fall in coming decades and some areas of India already have declining groundwater levels. To our knowledge, this study is the first to investigate the potential role of dietary modification as a solution to the increasing pressures on freshwater availability for agriculture in the coming decades.

**Added value of this study**

Optimisation modelling using India-specific food consumption and environmental data suggests that relatively minor dietary modifications could enable India to reduce per person freshwater requirements associated with irrigation in agriculture to 2025; larger changes to diets would be required to meet freshwater reduction targets to 2050. Our modelling demonstrates that the optimised diets that meet WHO nutritional guidelines would also deliver the co-benefits of reduced diet-related greenhouse gas emissions and improved population health.

**Implications of all the available evidence**

The Indian food system is likely to face increasing pressure throughout the 21st century to deliver healthy and nutritious diets as the population increases and groundwater availability declines. Agricultural improvements and technologies will contribute to meeting these challenges but dietary modifications that reduce the environmental impacts of Indian diets could also make substantial contributions to mitigation efforts.

## Methods

### Scenarios of future water availability

The Ministry of Water Resources estimates the current national annual average volume of available water in India at 1869 billion cubic metres (BCM).^3^ Accounting for hydrological and topological constraints, only 1123 BCM is considered to be useable.^3^ Current demand for irrigation is estimated to be 557 BCM per year,^3^ representing 49·6% of the total useable water. Due to projected growth in population, by 2050, demand for irrigation is expected to increase to more than 70% of usable water unless production technologies or diets change.^3^

We modelled changes to Indian dietary patterns under two time scenarios, accounting for population growth, that would maintain total water used for irrigation (blue water footprint) at the current level (557 BCM per year) by reducing water levels per person. Scenario one (the 2025 scenario): with projected population growth from 1·15 billion (2010) to 1·40 billion in 2025, water per person will be reduced by 18·0%.^3^ Scenario two (the 2050 scenario): with the population projected to reach 1·64 billion by 2050, water per person will be reduced by 30·3% compared with 2010.^3^ We accordingly reduced the average blue water footprints of Indian dietary patterns per person by 18·0% and 30·3% for the 2025 and 2050 scenarios, respectively.

### Dietary patterns and associated environmental effects

Our dietary patterns were based on the Indian Migration Study (IMS), a large and diverse cross-sectional survey of adult factory-employed urban migrants in Bangalore, Hyderabad, Lucknow, and Nagpur and their rural siblings (n=7067) from 2005 to 2007.^8^ Dietary intake was assessed using a semiquantitative food frequency questionnaire. We grouped IMS food items into 36 food groups based on compositional similarity and derived baseline average consumption (g/capita per day) for five distinct dietary patterns using finite mixture modelling. Briefly, the patterns were rice and low diversity—a rice-based pattern with low consumption of other food groups and relatively low total dietary energy; rice and fruit—a diet characterised by high consumption of rice and fruits; wheat and pulses—a diet containing higher than average levels of wheat, pulses, legumes, and vegetables but lower than average consumption of fruits; wheat, rice, and oils—a mixed pattern containing both wheat and rice and with the highest total dietary energy; and rice and meat—a rice-based pattern with greater than average consumption of meat and fish. Full details of the method and baseline dietary patterns are reported elsewhere.^9^

We linked each food group to data for blue water footprints and greenhouse gas emissions. The blue water footprint of crops represents the amount of water sourced from surface or groundwater resources used for irrigation during agricultural production.^10^ The blue water footprint of livestock products represents the water used to irrigate feed ingredients, and drinking and service water.^11^ Water footprint values were derived from the Water Footprint Network.^10^, ^11^, ^12^ The greenhouse gas emissions associated with agricultural production of crop and livestock products were estimated using the Cool Farm Tool based on farm-level activity data.^13^, ^14^ Emissions associated with food processing, transport, preparation, and waste were based on previous scientific literature.^15^ Detailed methods used to calculate water footprints and greenhouse gas emissions of IMS diets can be found in the appendix and elsewhere.^12^, ^14^

### Dietary modelling

We optimised each dietary pattern to derive new diets that reduced blue water use to meet the 2025 and 2050 targets on average across the five dietary patterns and achieved WHO nutritional guidelines for carbohydrates, fats, free sugars, protein, sodium, fruits, and vegetables (appendix)^15^ with no change in total dietary energy. The optimisation process minimised deviation from existing dietary patterns (deviation was calculated as the sum of squared percentage changes between baseline and optimised consumption in each food group; the percentage changes were squared to account for both increased and decreased consumption levels). To achieve the overall blue water use reduction across all dietary patterns, equitable targets for each of the five patterns were defined accounting for their baseline blue water footprints (ie, greater levels of reduction per person were required for dietary patterns with higher baseline footprints). In each scenario, the method converged blue water use per person on the same level for each dietary pattern, though with different contributions from each.

As a simplified measure of consumer preferences, each food group was weighted by the ratio of its share of dietary expenditure to its price elasticity (appendix) using published data for expenditure^16^ and price elasticities.^17^

The optimisations were done with R statistical software using the Alabama package, which uses an augmented Lagrangian method with an adaptive barrier function to optimise non-linear functions including constraints (the modelled constraints were requirements to reduce the diet's blue water footprint, to meet nutritional guidelines, and to maintain dietary energy). The output was a set of optimised dietary patterns with new levels of consumption for each food group.

### Modelling the health effects

We estimated the effect on mortality due to immediate adoption of each optimised dietary pattern using a life table method adapted from the IOMLIFET model coded in R.^18^ The model was set up using mortality and population data from the Indian Registrar General, the UN, and WHO, projected to 2050 by extrapolation of recent trends using second order polynomials.^19^, ^20^, ^21^, ^22^

Guided by evidence from the Global Burden of Disease study^23^ and a previous review of meta-analyses,^6^ we assessed the effect of changes in consumption of red meat (largely mutton in the Indian setting), fruits, and vegetables on the following outcomes: coronary heart disease, stroke, type 2 diabetes, and cancers of the mouth, pharynx or larynx, oesophagus, lung, stomach, and colon or rectum. Exposure-response functions were assumed to be log-linear and, where multiple exposures affected a single outcome, risks were assumed to be multiplicative. S-shaped curves (cumulative distribution functions of normally distributed variables), based on evidence on the effects of dietary interventions on mortality over time, were used to account for time lags in disease after dietary changes. The primary outcomes of the model were changes in life-years per 100 000 total population over 40 years (to 2050). Further details of the health impact model can be found in the appendix and previous reports.^24^

### Statistical analysis

We used a Monte Carlo simulation method with 1000 repetitions to obtain a measure of the variability associated with our estimates of optimised dietary changes and associated environmental and health effects. For each repetition, we sampled randomly from the distribution of input parameters, assuming normal distributions. For baseline consumption of each food group in each dietary pattern, we used the SDs of individual-level consumption for survey participants assigned to that pattern. For water footprints, variations were based on spatial differences reflecting state-level blue water footprints. For expenditure, estimates were based on the SEs of the survey data, and for exposure-response coefficients, we used 95% CIs from the original published sources. Where we were unable to obtain full information (nutritional composition, greenhouse gas emissions, and price elasticities), we assumed uniform distributions of plus or minus 10% around the central estimates. To reduce the likelihood of locating local minima, within each simulation, the optimisation was performed 20 times and the best result (minimum objective value while meeting all constraints) was selected.

### Role of the funding source

The funder of the study had no role in study design, data collection, data analysis, and data interpretation, or writing of the report. The corresponding author had full access to all the data in the study and had final responsibility for the decision to submit for publication.

## Results

The mean per person blue water footprints of the baseline dietary patterns ranged from 566 L/day (rice and low diversity) to 877 L/day (wheat, rice, and oils) and dietary greenhouse gas emissions ranged from 1·93 kilograms of carbon dioxide equivalents (kgCO_2_e)/day (wheat and pulses) to 3·33 kgCO_2_e/day (rice and meat; table 1). The optimised diets achieved the 2025 and 2050 blue water footprint reduction targets of 18·0% and 30·3%, respectively, across all patterns (table 2). These optimised diets generally featured increased consumption of fruits and vegetables and small increases from low baseline levels in mutton and other red meat consumption. Although the optimised diets remained distinct, common trends in the changes required to reduce overall blue water footprints were noted: large decreases in the amount of foods with the highest blue water footprints including wheat, dairy, eggs, poultry, nuts, and seeds; large increases in legumes (including red gram), which despite their relatively high blue water footprints are a good vegetable source of protein; switching from white meat (poultry) to mutton and other red meat with lower blue water footprints (largely due to the relatively lower water footprints of feed); and switching from fruits such as grapes, guava, and mango to those that have lower blue water footprints such as melon, orange, and papaya. Full details of the optimised diets can be found in the appendix.

**Figure 1:**
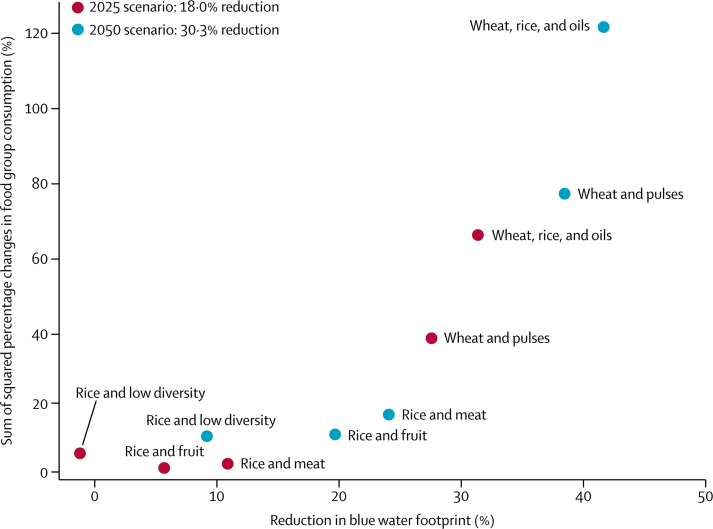
Mean deviations of optimised dietary patterns from current consumption (sum of squared percentage changes in consumption across all food groups) for different levels of blue water footprint reduction under 2025 and 2050 scenarios Percentage changes in consumption in each food group were squared to account for increases and decreases in consumption.

The total amount of water used for irrigation (557 BCM per year) remained unchanged after optimisation but, because of population growth, the per person blue water footprints of the diets were reduced (figure 1). The mean per person reductions were largest for wheat-based diets with high baseline levels: up to 38·5% (95% CI 36·0–40·6) in the wheat and pulses pattern and 41·7% (39·4–43·9) in the wheat, rice, and oils pattern by 2050 (table 2). Conversely, the blue water footprint of the rice and low diversity pattern increased slightly in the 2025 scenario (to achieve the overall reduction across all dietary patterns) but decreased for the more stringent 2050 target (9·2%, 95% CI 3·4–14·7). The rice and fruit and rice and meat patterns were in the mid range of water footprint reductions.

The changes in dietary patterns following optimisation were accompanied by reductions in dietary greenhouse gas emissions. There were particularly large greenhouse gas reductions in the wheat and pulses pattern of up to 36·2% (95% CI 19·4–49·1) by 2050, due to large declines in dairy products. Changes in dietary greenhouse gas emissions in the other patterns were smaller. No simulations in the Monte Carlo analysis showed increased emissions (appendix).

**Figure 2:**
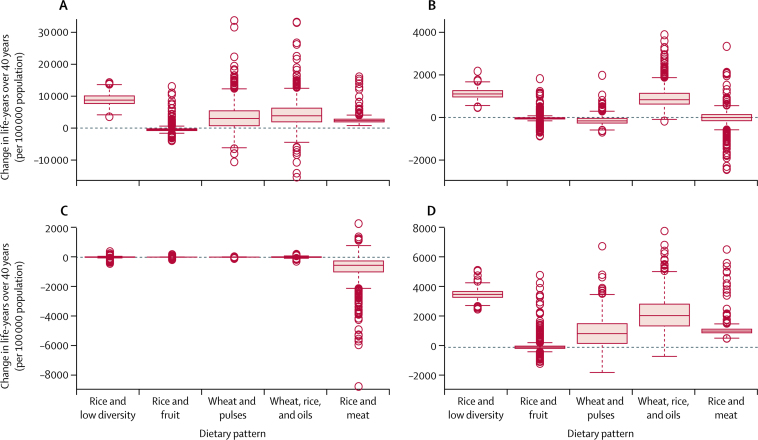
Modelled health impacts (changes in life-years over 40 years per 100 000 population) for each dietary pattern under the 2025 scenario as a result of adoption of optimised Indian dietary patterns for coronary heart disease (A), stroke (B), type 2 diabetes (C), and cancers (D) Positive values indicate health benefits. Thick lines indicate the median, boxes indicate the IQR, whiskers indicate the limits of nominal range, and open circles indicate outliers.

**Figure 3:**
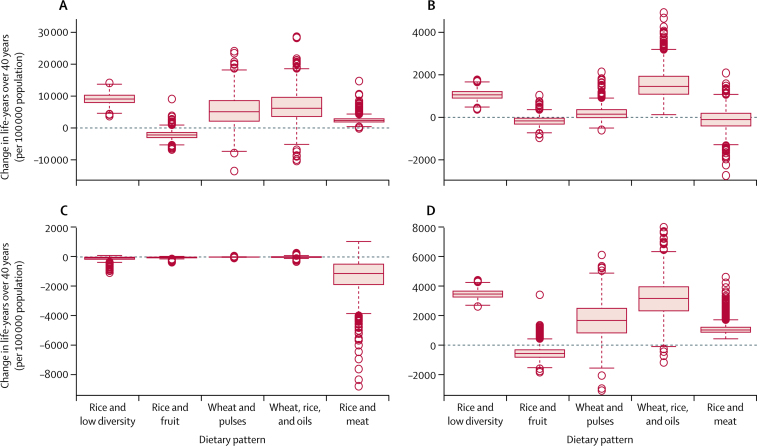
Modelled health impacts (changes in life-years over 40 years per 100 000 population) for each dietary pattern under 2050 scenario as a result of adoption of optimised Indian dietary patterns for coronary heart disease (A), stroke (B), type 2 diabetes (C), and cancers (D) Positive values indicate health benefits. Thick lines indicate the median, boxes indicate the IQR, whiskers indicate the limits of nominal range, and open circles indicate outliers.

If these optimised diets were to be adopted within our studied Indian population, our model suggests that this would be broadly beneficial for health. Estimated health impacts under the two scenarios were relatively similar (Figure 2, Figure 3). Generally, the low blue water footprint diets would provide benefits for coronary heart disease, stroke, and cancer, of which the largest benefits would be for coronary heart disease. However, because of the switch from white meat to mutton and other red meat (especially in the rice and meat pattern), there would be some increase in the risk of type 2 diabetes.

In four of the five patterns, after optimisation the mean effect combined across all health outcomes would be positive (appendix). For example, under the 2050 scenario, the net effect across all patterns was 6800 life-years gained per 100 000 population (95% CI 1600–13 100) over the 40-year follow-up period (rounded to the nearest hundred). The health benefits were largest in the rice and low diversity (13 500, 10 200–16 800) and wheat, rice, and oils (11 400, 800–26 300) patterns. The exception was the rice and fruit pattern that contained high levels of fruit consumption at baseline; reduced fruit consumption in the rice and fruit pattern would lead to increased risk of coronary heart disease, stroke, and some cancers, especially when vegetable consumption was also reduced in the 2050 scenario, resulting in 2800 life-years lost per 100 000 population (–1500 to −6300) over 40 years.

The Monte Carlo analysis shows that some combinations of variability in the input parameters can result in overall adverse effects on health (Figure 2, Figure 3; appendix). However, broadly speaking, our results suggest that consumption of low blue water footprint diets would improve health in the study population.

## Discussion

We show that modest changes to dietary patterns could reduce irrigation water requirements per person in a geographically diverse Indian sample and that these changes would also reduce diet-related greenhouse gas emissions and improve population health. These multiple benefits highlight the importance of dietary change as a means to tackle planetary health challenges. The broader picture is, however, more complex as some required dietary changes by 2050 would be relatively large (figure 1); some are contrary to current trends in diets; and some might have adverse effects on population health and nutrient intake (appendix).

Our study is theoretical and the results should be viewed as indicative. Our scenarios were based on projections of population growth only and did not account for other potential drivers with more complex and uncertain effects, such as climate change and aquifer depletion. We also did not account for non-food crop production, current dietary trends, temporal variation in water availability, or the effects of socioeconomic differences between dietary patterns. Overall, our scenarios are likely to be conservative. Estimates of water availability and irrigation demand in India vary substantially, with some predicting supply to fall to only 50% of demand by 2030,^25^ although the estimates used for our scenarios are broadly consistent with others.^26^

Our dietary patterns were derived from a large and diverse population sample, but are not nationally representative. In particular, the IMS over-represents wealthier, urban dwellers relative to India as a whole and the sample does not include children. Our analysis also did not account fully for regional variations in water footprints, although these were included implicitly in the distributions of blue water footprints used as input to the model. Additionally, we could not distinguish between renewable and non-renewable groundwater or consider effects on green (rainfall) water use. Under climate change, rainfall is likely to become increasingly unpredictable in India,^27^ providing additional rationale for ensuring sustainable ground and surface water use. We also did not account for the potential effects of poor water quality on availability for irrigation,^28^ nor the additional strain on water resources from the need to produce more and diverse foods for malnourished population groups. There are also uncertainties in our estimation of health effects as we assumed instantaneous adoption of the optimised diets and we only included a limited number of health outcomes to avoid potential double counting and confounded associations.

Studies on modification of diets to reduce greenhouse gas emissions and improve health are increasing,^5^ but few previous studies have attempted to optimise diets to reduce water use. A multi-objective method used to optimise diets in Italy found that a sustainable diet (integrating environmental and economic sustainability) used the least water.^7^ To date, studies have used only stochastic methods to examine uncertainties. To our knowledge, this study is the first of its type to combine dietary optimisation with an assessment of potential variability, albeit with simplifying assumptions because of data limitations, and we provide an indication of the scale of probable variability in our estimates. An improved understanding of uncertainties is key for designing appropriate policies and interventions; our Monte Carlo simulations identified some potential adverse effects on health that would not have been captured by standard deterministic methods.

Future research on the nature and implications of sustainable and healthy diets will need to account for changing consumption trends, including transitions to more energy-dense and processed foods, that will have consequences for health, water availability, greenhouse gas emissions, and land use.^4^ The effect of environmental change on food systems through changes in rainfall patterns, water availability, and crop yields, although uncertain and likely to vary regionally, must also be better understood.^29^ In India, for example, monsoon precipitation, which is projected to become more variable in the future as a result of climate change, has been shown to affect groundwater storage but with large geographical differences.^30^ From a policy perspective, our study focused only on demand-side solutions: realistic changes in a set of typical dietary consumption patterns. Policy action to tackle the effects of reduced water availability on food production could also exploit a range of supply-side opportunities that remain largely unexplored, including improved agronomic and irrigation technologies, taxation, and international trade (including food imports).

Despite these tensions and potential trade-offs, this study shows that, at least over coming decades, Indian dietary patterns could be modified in line with projected per person reductions in water availability in a way that protects the environment and enhances the health of the population. These changes could play an important part in the development of a resilient food system in India.

## Figures and Tables

**Table 1:** Mean per person characteristics of baseline dietary patterns

	**Energy intake (kcal/day)**	**Blue water footprint (L/day)**	**Greenhouse gas emissions(kgCO_2_e/day)**
Rice and low diversity	2369	566	2·91
Rice and fruit	2762	640	2·73
Wheat and pulses	3027	836	1·93
Wheat, rice, and oils	3344	883	2·06
Rice and meat	2723	677	3·33

**Table 2:** Key dietary changes and associated environmental effects in optimised Indian dietary patterns for 2025 and 2050 scenarios

	**Changes in dietary consumption (g/day)**				**Change in environmental impact**	
	Fruits	Vegetables	Mutton and other red meat	Poultry*	Blue water footprint	Greenhouse gas emissions
**2025 scenario: minimum 18·0% blue water footprint reduction**						
Rice and low diversity	95·2 (85·1 to 100·7)	36·6 (30·8 to 48·4)	0·3 (–0·8 to 3·1)	2·4 (–1·7 to 15·3)	1·2% (7·1 to −1·4)	–3·4% (–0·0 to −9·3)
Rice and fruit	–1·1 (–23·3 to 23·8)	–1·6 (–7·3 to 10·4)	0·2 (–0·2 to 1·4)	–7·1 (–0·8 to −13·4)	–5·7% (–1·4 to −9·7)	–2·5% (–0·0 to −5·4)
Wheat and pulses	–9·5 (–32·8 to 23·7)	42·5 (–22·3 to 116·4)	0·1 (–0·2 to 0·5)	–6·0 (–4·8 to −6·7)	–27·6% (–24·8 to −30·3)	–29·5% (–13·3 to −45·8)
Wheat, rice, and oils	78·1 (16·5 to 161·6)	–2·6 (–73·4 to 57·6)	0·1 (–2·4 to 2·0)	–10·7 (–9·0 to −12·3)	–31·4% (–28·7 to −33·8)	–3·2% (–0·0 to −16·9)
Rice and meat	23·0 (15·2 to 30·8)	12·4 (7·2 to 17·7)	18·4 (–6·9 to 59·1)	–21·8 (–12·2 to −27·6)	–10·9% (–6·2 to −15·2)	–1·7% (–0·0 to −6·1)
Weighted average	34·4 (13·1 to 64·1)	19·5 (–13·4 to 52·4)	1·5 (–0·6 to 5·3)	–6·8 (–3·0 to −9·1)	–18·8% (–18·0 to −19·8)	–8·9% (–4·4 to −16·1)
**2050 scenario: minimum 30·3% blue water footprint reduction**						
Rice and low diversity	90·1 (74·3 to 99·9)	41·5 (31·8 to 57·3)	2·6 (–0·4 to 8·5)	–7·6 (–14·7 to 5·8)	–9·2% (–3·4 to −14·7)	–3·5% (–0·0 to −10·5)
Rice and fruit	–11·1 (–40·6 to 27·4)	–13·6 (–29·1 to 2·1)	1·1 (–0·2 to 3·8)	–16·1 (–14·1 to −17·3)	–19·7% (–16·1 to −23·3)	–10·1% (–2·1 to −17·8)
Wheat and pulses	15·9 (–21·0 to 73·5)	49·7 (–33·5 to 132·4)	0·2 (–0·2 to 0·7)	–6·0 (–5·3 to −6·7)	–38·5% (–36·0 to −40·6)	–36·2% (–19·4 to −49·1)
Wheat, rice, and oils	134·6 (41·4 to 291·8)	–11·5 (–96·7 to 65·1)	0·2 (–2·7 to 2·7)	–10·7 (–9·0 to −12·4)	–41·7% (–39·4 to −43·9)	–4·3% (–0·0 to −19·3)
Rice and meat	33·1 (15·2 to 82·6)	5·5 (–11·7 to 19·3)	32·3 (–11·7 to 79·0)	–25·9 (–23·4 to −28·3)	–24·1% (–20·4 to −27·9)	–5·2% (–0·0 to −14·0)
Weighted average	51·5 (17·0 to 105·3)	17·5 (–22·2 to 54·4)	3·3 (–0·6 to 7·8)	–6·8 (–3·0 to −9·1)	–30·3% (–30·3 to −30·3)	–12·9% (–6·7 to −20·5)

Data are mean change (95% CI) based on Monte Carlo simulation.

* Not included in health impact model.
