# Supplementary material for: Projected health effects of realistic dietary changes to address freshwater constraints in India: a modelling study
Source: Lancet Planet Health. 2017 Apr;1(1):e26–32. doi: 10.1016/S2542-5196(17)30001-3 (PMC5408829; doi:10.1016/S2542-5196(17)30001-3)
Supplement: Supplementary appendix [file mmc1.pdf]

THE LANCET
Planetary Health

Supplementary appendix

This appendix formed part of the original submission and has been peer reviewed. We post it as supplied by the authors.

Supplement to: Milner J, Joy EJM, Green R, et al. Projected health effects of realistic dietary changes to address freshwater constraints in India: a modelling study. *Lancet Planetary Health* 2017; **1:** e26–32.

Supplementary material

Projected health effects of realistic dietary changes to address freshwater constraints in India: a modelling study

INTRODUCTION

This appendix accompanies the paper "Projected health effects of realistic dietary changes to address freshwater constraints in India: a modelling study". It provides additional details on the methods used and shows results not presented in the main paper.

METHODS

We optimized a set of typical dietary patterns in India to meet projected decreases in *per capita* water availability (based on population growth) while remaining as close as possible to existing dietary patterns. The health impacts that would result from each dietary shift were modelled using life tables. Changes in resulting dietary greenhouse gas (GHG) emissions were estimated as an ancillary outcome. We used a Monte Carlo approach to assess variability in the results.

Scenarios of future water availability in India

The Ministry of Water Resources estimates the current national annual average volume of available water in India at 1869 billion cubic metres (BCM).[1] Accounting for hydrological and topological constraints, only 1123 BCM is considered to be utilizable.[1] Current demand for irrigation is estimated to be 557 BCM per year,[1] representing 49.6% of the total utilizable water. Due to projected growth in population and irrigation demand, by mid-century demand for irrigation is expected to increase to more than 70% of utilizable water.[1]

We modelled changes to Indian dietary patterns under two time scenarios, accounting for population growth, which would maintain total water used for irrigation (blue water footprint) at the current level (557 BCM per year) by reducing *per capita* levels:

1) 2025 scenario: By 2025, with projected population growth from 1·15 billion (2010) to 1·40 billion, *per capita* water will be reduced by 18·0%.[1]
2) 2050 scenario: The population of India is expected to reach 1·64 billion by 2050, resulting in a 30·3% reduction in *per capita* water compared to 2010.[1]

We accordingly reduced the average *per capita* blue water footprints of Indian dietary patterns by 18·0% and 30·3% for the 2025 and 2050 scenarios, respectively.

Identification of baseline dietary patterns

The work was based on analysis of the Indian Migration Study (IMS), a cross-sectional survey of factory-employed urban migrant adults in Bangalore, Hyderabad, Lucknow and Nagpur and their rural siblings (n=7067) between 2005 and 2007.[2] As part of the IMS, dietary intake was assessed using a semi-quantitative food frequency questionnaire (FFQ).[3] To characterise Indian diets, we derived the nutritional composition (including total energy and levels of carbohydrate, fats, protein, vitamins and various micronutrients) of each of the survey's 199 food items using Indian food composition tables and, where local data were unavailable, US composition tables.[4,5] We grouped the IMS food items into 36 food groups based on compositional similarity (Table S1).

Table S1. Classification of IMS food items into 36 food groups.

Food group	Individual food items
Cereals (other)	Bajra (pearl millet); Bajre ki atta; Bhagar (wild grass seed); Ragi (millet); Ragi flour; Corn flour; Jowar (sorghum) flour; Cornflakes
Rice	Rice (polished); Rice flakes; Puffed rice; Rice flour
Wheat	Noodles; Dalia; Rava; Wheat flour; Bread
Butter/ghee	Butter; Ghee
High fat dairy	Paneer; Khoa; Cream; Ice cream
Low fat dairy	Curds; Hung curd; Milk
Egg	Egg
Fish/seafood	Fish; Prawn
Banana	Banana
Grapes	Grapes
Guava	Mango
Mango	Raw mango
Melon	Musk melon; Watermelon
Orange	Orange
Fruit (other)	Apple; Raw plantain; Amla; Custard apple; Raisins; Jack fruit; Kiwi; Dry mango slice; Lemon; Lemon juice; Sweet lime; Lime juice; Litchis; Palmyra; Peaches; Pears; Pineapple; Plums; Pomegranate; Sapota; Tamarind; Zizyphus; Jamoon; Coconut; Copra; Coconut milk
Papaya	Papaya
Leafy vegetables	Amaranth; Cabbage; Green vegetable (dhantu); Gogu; Spinach; Corriander leaves; Mint leaves
Legumes	Kidney beans; Cluster beans; Groundnut
Mutton	Mutton
Meat (other)	Liver; Brain; Pigeon; Rabbit; Salami;
Poultry	Chicken; Other poultry
Nuts/seeds	Almonds; Cashewnuts; Chironji; Sesame seeds; Pistachio nut
Other	Chocolate; Baking powder; Beer; Spirits (whiskey, gin, rum); Coca-cola; Soda; Coffee powder; Custard powder/corn flour; Horlicks; Jam; Kala namak; Ketchup/tomato sauce; Lemon pickle masala; Local arrack/toddy; Mango pickle masala; Papad khar; Pav; Pizza; Tea powder; Sago; Soya sauce; Wine; Vinegar
Other (sugar)	Sugarcane; Sugar; Honey; Jaggery
Potato	Potato
Pulses (other)	Chick peas; Besan; Bengal gram dhal; Black gram; Black gram dhal flour; Black gram dhal; Peas; Green gram; Green gram dal; Masoor dhal; Semi falli (broad bean)
Redgram	Red gram dhal
Salt	Salt
Spices (other)	Kamal kakdi; Omum; Ajwain powder; Amchur powder; Asofoetida; Bay leaves; Black cardamom; Biriyani powder; Pepper corn; Cardamom; Cardamom powder; Cinnamon; Cloves; Red chilli; Dry red chilli; Red chilli powder; Ginger; Coriander seeds; Jeera; Cumin seed powder; Curry leaves; Sambar powder; Saunf; Methi seeds; Kasuri methi; Jaiphal; Javithri; Mustard; Mustard powder; Onion seed; Poppy seeds; Shajeera; Turmeric powder; Danya powder; Ansi flower
Starchy roots	Colacasia; Yam
Carrot	Carrot
Gourd	Bitter gourd; Bottle gourd; Dhemsa/tinda; Lauki; Kundru; Parwal; Ridge gourd
Vegetables oils	Dalda; Sunflower oil; Mustard oil; Soya oil; Groundnut oil; Palm oil; Rice bran oil; Coconut oil; Olive oil
Onion/garlic	Green onion; Garlic; Onion; Spring onions
Vegetables (other)	Green beans; Brinjal; Capsicum; Cauliflower; Cucumber; Drum stick; Ladies finger (okra); Mushroom; Red pumpkin; Beetroot; Radish; Turnip; Chow chow marrow; Green chilli
Tomato	Tomatoes; Tomato puree

Distinct dietary patterns were derived using finite mixture modelling. The 36 food groups (consumption variables) were entered into a Latent Class Analysis (LCA) model to identify distinct patterns of food consumption based on clustering in the data.[6] Solutions containing 1–10 distinct dietary patterns were specified, and we used a combination of diagnostic criteria (Bayesian Information Criterion, minimum proportion per class and Entropy of model) to select the solution that fitted the data best. The five-pattern model provided the best fit to the data, and survey individuals were assigned to one of these five dietary patterns based on their probability of inclusion in each pattern. The final patterns were expressed as average consumption in each food group in g/capita/day. Full details of the method and the final dietary patterns can be found in other work by the authors.[7]

Environmental impacts of diets

Each food group was linked to data on its respective blue water footprint and GHG (CO_2e) emissions as follows:

Water footprints

Crop items were matched to state level crop blue water footprints obtained from Mekonnen and Hoekstra (2011).[8] The blue water footprints of animal products were calculated following methods described in Mekonnen and Hoekstra (2012),[9] which estimates water footprints from the indirect water footprint of feed and the direct water consumption from drinking and service water. The animal categories assessed were: beef and dairy cattle, pig, sheep, goat, broiler and layer chicken, and the water footprint was calculated for each of grazing, mixed and industrial systems. The ratio of different production systems for India was obtained from work by Wint and Robinson (2007).[10] The volume of feed required for each animal category was calculated using the feed conversion efficiency and the animal product output. Feed composition was estimated from FAO Supply and Utilisation accounts and work by Steinfeld and colleagues.[11] The water footprints of the feed were estimated using Indian state-level data on the water footprints of feed components (concentrate and roughage) and the additional water used for mixing (blue). This was combined with drinking and service water use (m^3/animal/day), and converted to m^3/tonne of product using Indian data on animal weight and yield (FAO, 2003).[12] Finally, product and value fractions taken from Mekonnen and Hoekstra (2012)[9] were used to convert into consumable animal product. For prawns and fish, the water footprints were estimated using the conversion factors per edible product,[13] the total feeds used for each, and the composition of feeds for each, based on the state-level crop data. The state-level data was then weighted by land-size using information from the Indian census,[14] and a standard error of the mean used to indicate variation for the Monte Carlo simulation (see below). Full details of the methods used to calculate water footprints of food items are reported elsewhere.[15]

GHG emissions

Emissions of GHGs across the life cycle (kg CO_2e/kg food) for each of the 36 food groups were derived from published data. Where possible, India-specific data were used, but where these were not available for particular food groups, data were extrapolated from other foods and/or countries. For each group, we combined estimates of emissions from food production, storage, processing, transport, cooking and packaging.[16–22] An additional factor for emissions due to food waste was added, which was quantified as the product of emissions from all other stages and the proportion of different food groups typically wasted at all stages from production to consumption using FAO estimates for South and South East Asia.[23] The GHG emissions associated with the 20 most-consumed crop and livestock products up to the production stage were estimated using a modified version of the Cool Farm Tool,[24] based on farm-level activity data for India.[25] These were supplemented with emissions for the remaining 16 food groups, as well as those for the processes from farm gate to consumer based on previous literature.[26]

Dietary optimization modelling

We optimized each dietary pattern to derive new diets which (i) reduced blue water use to meet the 2025 and 2050 targets on average across the five dietary patterns and (ii) achieved World Health Organization (WHO) nutritional guidelines for carbohydrates, fats, free sugars, protein, sodium, fruits, and vegetables (Table S2)[27] with minimal deviation from existing intake (sum of squared percentage differences for all food groups) and no change in total dietary energy.

Table S2. WHO nutritional guideline values.

Nutrient / food group	WHO guideline
Total fat (% total energy)	15–30%
Saturated fat (% total energy)	<10%
Polyunsaturated fat (% total energy)	6–10%
N6 polyunsaturated fat (% total energy)	5–8%
N3 polyunsaturated fat (% total energy)	1–2%
Trans fat (% total energy)	<1%
Monounsaturated fat (% total energy)	(remaining)
Carbohydrate (% total energy)	55–75%
Free sugars (% total energy)	<10%
Protein (% total energy)	10–15%
Sodium (g)	<2 g
Fruit and vegetables (g)	≥400 g

To achieve the overall blue water use reduction across all dietary patterns, equitable targets for each of the five patterns were defined accounting for their baseline blue water footprints (i.e. greater levels of *per capita* reduction were required for dietary patterns with higher baseline footprints). In each scenario, the method converged *per capita* blue water use on the same level for each dietary pattern, though with different contributions from each.

In the optimization, for a given food group i, the loss of welfare W_i resulting from consumption greater or less than the ideal level for health is proportional to the share of expenditure for that food group s_i and inversely proportional to the price elasticity of demand ε_i

$$\Delta W_i \propto \frac{s_i}{\varepsilon_i}\left(\frac{\Delta X_i}{X_i}\right)^2$$

where X_i is the current consumption for food group i and ΔX_i is the difference between current and ideal consumption for food group i. The ratio of s_i/ε_i acts as a simplified measure of utility. Data on the share of dietary expenditure (s_i) were taken from the nationally-representative National Sample Survey, conducted in 2004–2005.[28] The survey recorded the quantity and value purchased in the last 30 days of a comprehensive list of about 250 food and beverage items. Expenditure share for each of the 36 food groups was calculated by dividing the value spent on a food group by total expenditure on all food. Data on price elasticities for each food group (ε_i) were taken from work by Kumar and colleagues.[29]

The analysis then seeks to find the combination of foods that minimizes the weighted deviations of squared percentage consumption from the desired levels, where each deviation is weighted by s_i/ε_i. For the 36 food groups in the analysis, this can be expressed as

$$\min_{\{\Delta X_i; i=1..36\}} \left[\sum_{i=1}^{36} \frac{s_i}{\varepsilon_i}\left(\frac{\Delta X_i}{X_i}\right)^2\right]$$

Initial estimates of optimized consumption for each food group (i.e. initial estimates of the solution of the above equation) were generated randomly. The optimization was performed in the statistical language R[30] using the package Alabama, which uses an augmented Lagrangian method with an adaptive barrier function to optimize nonlinear functions including constraints.[31] The augmented Lagrangian method is a form of nonlinear programming that works by replacing the (constrained) problem with a sequence of unconstrained functions that are augmented with a penalty function. Lagrange multipliers (commonly used in mathematical optimization) are used to find the local minima or maxima at each stage. The application of the method to dietary optimization has been described in previous work by the authors.[32,33]

Modelling the impact on health

We estimated the impact on mortality due to adoption of each optimized dietary pattern using a life table method adapted from the IOMLIFET model[34] coded in R. The model was set up using age- and cause-specific mortality and population data from the Indian Registrar General, United Nations and WHO projected to 2050 based on extrapolation of recent trends using second order polynomial functions.[35–38]

Guided by evidence from the Global Burden of Disease (GBD) study[39] and a previous literature review of meta-analyses relating food or nutrient consumption to non-communicable disease,[32] we assessed the impact on health through the effects of changes in consumption of mutton and other red meat, fruits, and vegetables (Table S3) on the following mortality outcomes: coronary heart disease, stroke, type 2 diabetes and cancers of the mouth/pharynx/larynx, oesophagus, lung, stomach, and colon/rectum (Table S4).

Table S3. Food groups classified as mutton and other red meat, fruits, and vegetables.

Dietary exposure	Food groups
Mutton and other red meat	Mutton; Meat (other)
Fruits	Banana; Grapes; Guava; Mango; Melon; Fruit (other); Orange; Papaya
Vegetables	Carrot; Gourd; Leafy vegetables; Onion/garlic; Vegetables (other); Tomato

Table S4. Underlying cause of death classifications (ICD-10) used for each health outcome.

Health outcome	ICD-10 underlying cause of death classification	
	Codes	Underlying causes
Coronary heart disease	I20–I25	Ischaemic heart diseases
Stroke	I61–I64	Intracerebral haemorrhage; Other nontraumatic intracranial haemorrhage; Cerebral infarction; Stroke not specified as haemorrhage or infarction
Mouth/pharynx/larynx cancer	C00–C10, C12–C14, C32	Malignant neoplasms of lip, oral cavity and pharynx (excluding Malignant neoplasm of nasopharynx)*; Malignant neoplasm of larynx
Oesophageal cancer	C15	Malignant neoplasm of oesophagus
Lung cancer	C33–C34	Malignant neoplasm of trachea, bronchus and lung
Stomach cancer	C16	Malignant neoplasm of stomach
Colon/rectal cancer	C18–C20, C21.8	Malignant neoplasm of colon; Malignant neoplasm of rectosigmoid junction; Malignant neoplasm of rectum; Overlapping lesion of rectum, anus and anal canal[+]
Type 2 diabetes	E11	Non-insulin-dependent diabetes mellitus

* Malignant neoplasm of nasopharynx (ICD-10 C11) excluded since this was considered separately in original analysis
\+ Overlapping lesion of rectum, anus and anal canal (ICD-10 C21.8) included for consistency with Cancer Research UK (http://www.cancerresearchuk.org/cancer-info/cancerstats/types/bowel/survival/bowel-cancer-survival-statistics)

The exposure-response functions for each pathway, taken from previous meta-analyses,[40–44] were assumed to be log-linear and, where multiple exposures affected a single outcome, the risks were assumed to be multiplicative (Table S5).

Table S5. Dietary exposure-response pathways used in health impact model

Dietary exposure	Health outcome	Relative risk (95% confidence intervals)	Source
Fruit	Coronary heart disease	0·93 (0·89–0·96) per 80 g increase per day	Dauchet et al. (2006)[40]
	Stroke	0·89 (0·85–0·93) per 80 g increase per day	Dauchet et al. (2005)[41]
	Mouth/pharynx/larynx cancer	0·72 (0·59–0·87) per 100 g increase per day	Marmot et al. (2007)[42]
	Oesophagus cancer	0·56 (0·42–0·74) per 100 g increase per day	Marmot et al. (2007)[42]
	Lung cancer	0·94 (0·90–0·97) per 80 g increase per day	Marmot et al. (2007)[42]
	Stomach cancer	0·67 (0·59–0·76) per 100 g increase per day	Marmot et al. (2007)[42]
Vegetables (non-starchy)	Coronary heart disease	0·89 (0·83–0·95) per 80 g increase per day	Dauchet et al. (2006)[40]
	Stroke	0·97 (0·92–1·02) per 80 g increase per day	Dauchet et al. (2005)[41]
	Mouth/pharynx/larynx cancer	0·72 (0·63–0·82) per 50 g increase per day	Marmot et al. (2007)[42]
	Oesophagus cancer	0·87 (0·72–1·05) per 50 g increase per day	Marmot et al. (2007)[42]
	Stomach cancer	0·70 (0·62–0·79) per 100 g increase per day	Marmot et al. (2007)[42]
Mutton and other red meat	Colon/rectal cancer	1·29 (1·04–1·60) per 100 g increase per day	Marmot et al. (2007)[42]
	Type 2 diabetes	1·19 (1·04–1·37) per 100 g increase per day	Pan et al. (2011)[43]
	Stroke	1·21 (1·10–1·33) per 100 g increase per day	Micha et al. (2010)[44]

S-shaped curves (based on cumulative distribution functions of normally distributed variables) were used to account for the time lags in disease following changes in dietary exposure. The shapes of these functions were determined by evidence on the effects of dietary interventions on mortality over time.[45–48] The assumed lags for coronary heart disease, stroke, and type 2 diabetes reach a maximum impact after approximately 10 years (Figure S1) and for cancers after around 30 years, with no change in cancer risk for the first 10 years (Figure S2).

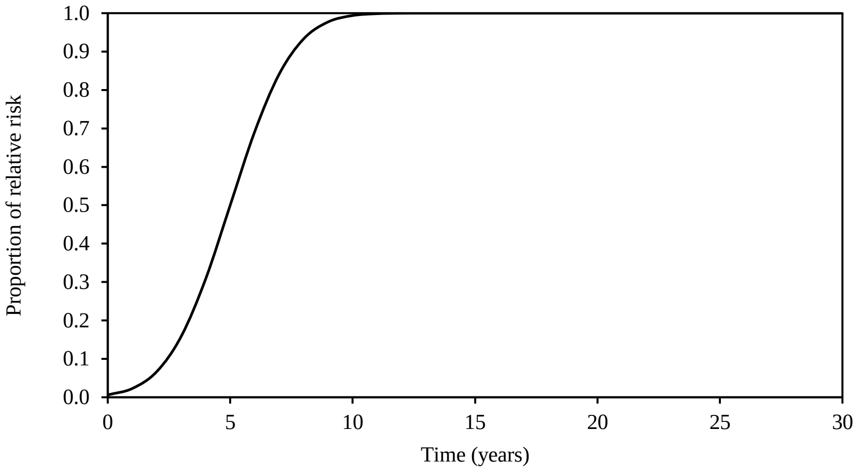

Figure S1. Time lag function used for coronary heart disease, stroke and type 2 diabetes.

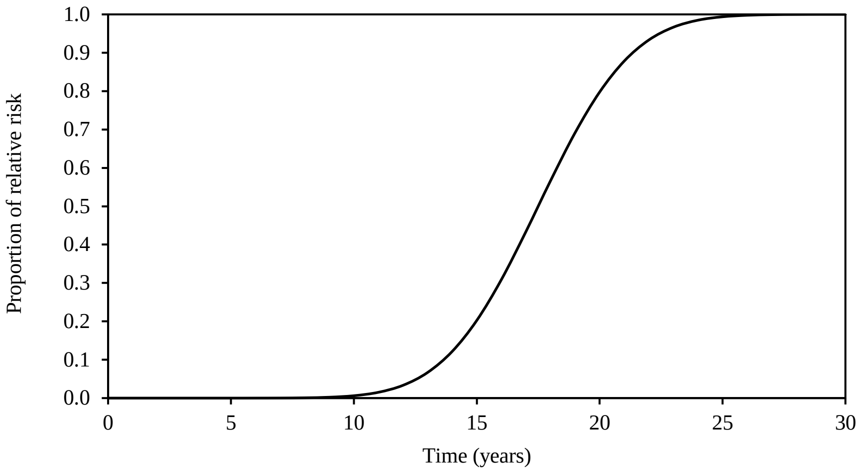

Figure S2. Time lag function used for cancer outcomes.

Further details of the health impact model, including time lag functions, can be found in previous work by the authors.[33] The primary outcomes of the model were changes in life years lived due to each outcome over a follow up period of 40 years (i.e. to 2050).

Monte Carlo analysis

We employed a Monte Carlo method whereby each simulation was repeated 1000 times to obtain a measure of the uncertainties associated with our estimates. For each repetition, we sampled randomly from the distribution of input parameters (water footprints, expenditure shares, exposure-response coefficients), assuming normal distributions for each. For the baseline consumption in each dietary pattern, we took consumption of each food group for each individual in the IMS data assigned to that pattern and estimated the standard deviations. For water footprints, the level of variation was based on spatial differences in the state-level data, which were dependent on differences in yields and climate factors. For expenditure shares, the estimates were based on the standard errors of the survey data and, for the exposure-response coefficients, we used the 95% confidence intervals from the original published sources. Where we were unable to obtain full information on the uncertainties (nutritional composition, GHG emissions, price elasticities), we assumed uniform distributions of ±10% around the central estimates. To reduce the likelihood of locating local minima, within each individual simulation the optimization process was repeated 20 times and the ‘best’ result (minimum objective value while meeting all constraints) was selected.

RESULTS

Consumption in baseline and optimized dietary patterns: 2025 scenario

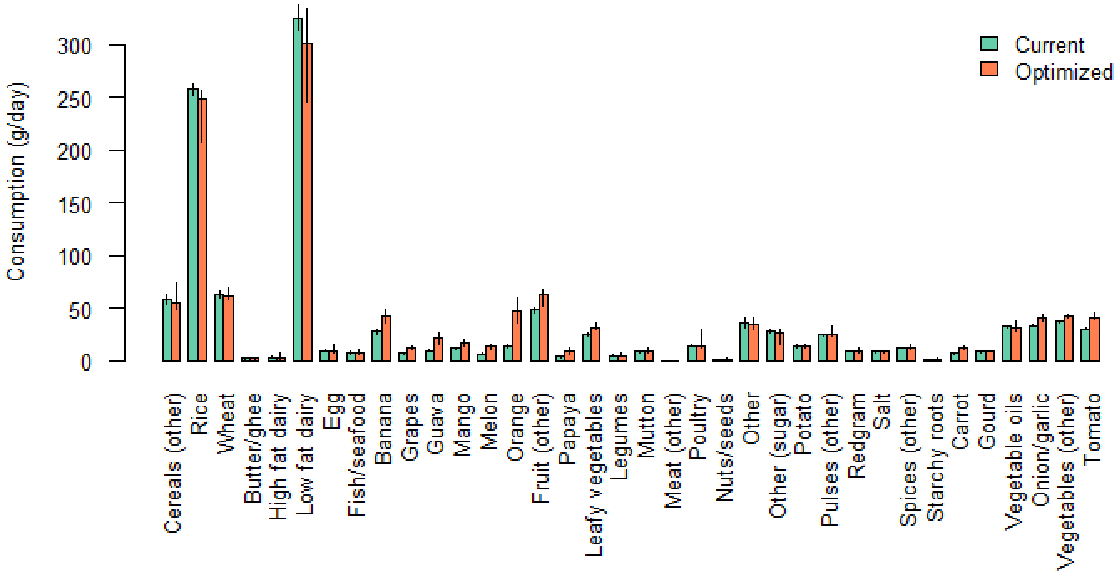

Figure S3. Average consumption of 36 food groups in g/day for *Rice and low diversity* pattern currently (green bars) and following optimization (orange bars) under 2025 scenario. Error bars = 95% confidence intervals.

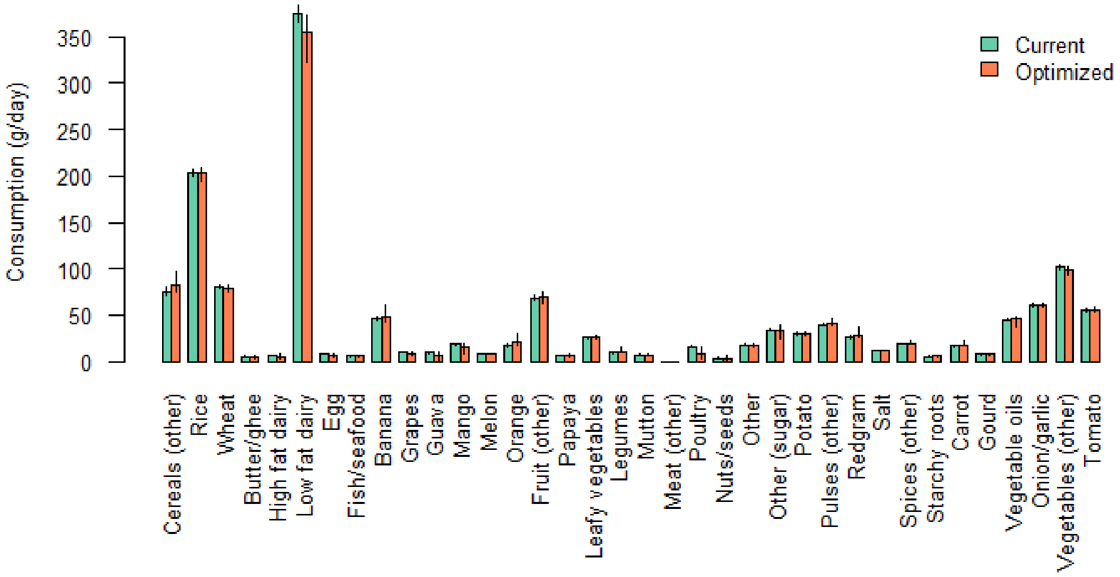

Figure S4. Average consumption of 36 food groups in g/day for *Rice and fruit* pattern currently (green bars) and following optimization (orange bars) under 2025 scenario. Error bars = 95% confidence intervals.

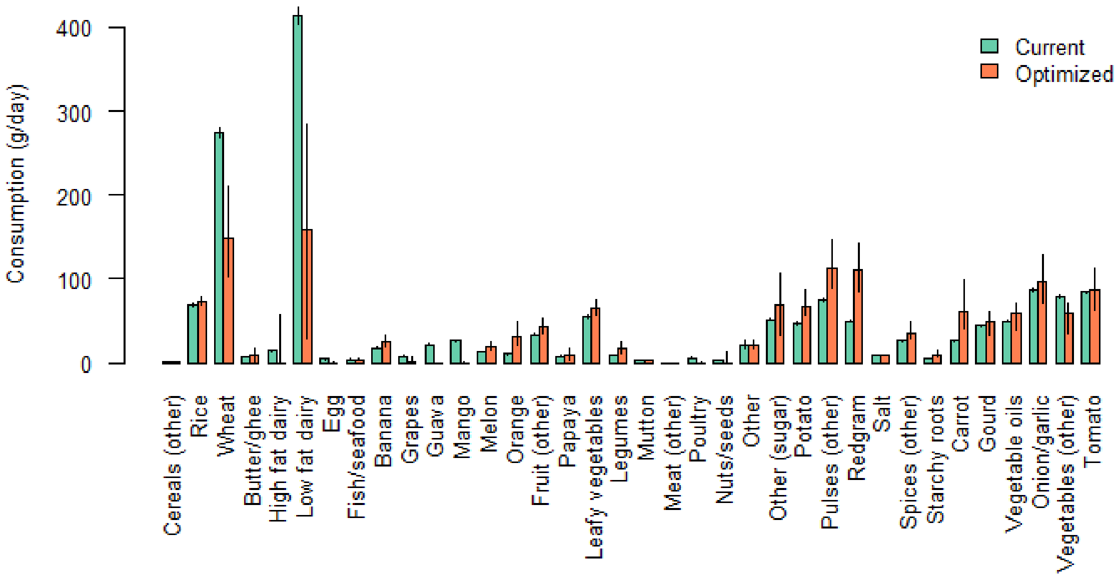

Figure S5. Average consumption of 36 food groups in g/day for *Wheat and pulses* pattern currently (green bars) and following optimization (orange bars) under 2025 scenario. Error bars = 95% confidence intervals.

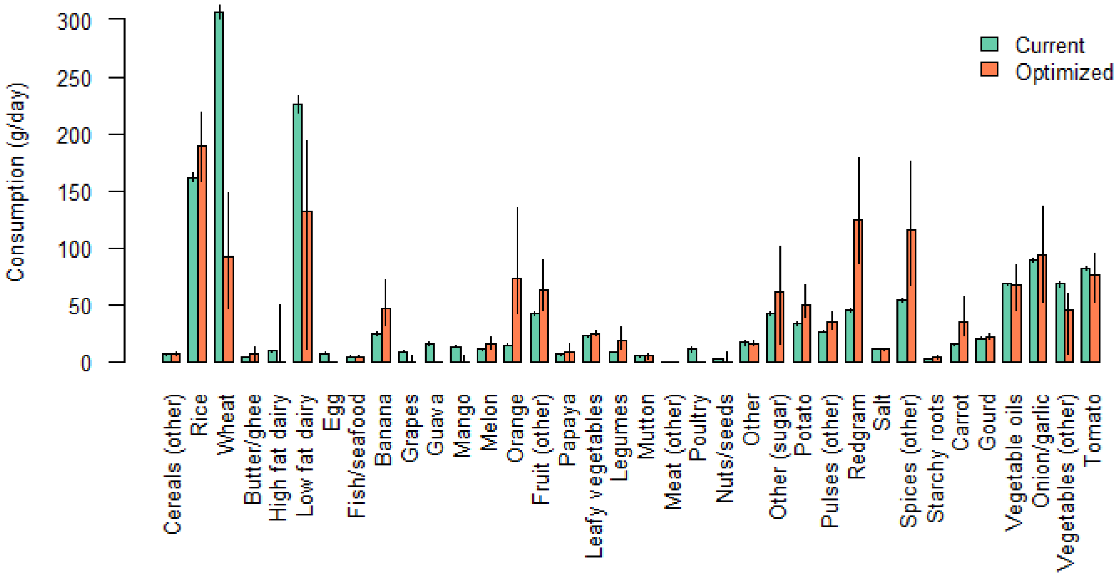

Figure S6. Average consumption of 36 food groups in g/day for *Wheat, rice and oils* pattern currently (green bars) and following optimization (orange bars) under 2025 scenario. Error bars = 95% confidence intervals.

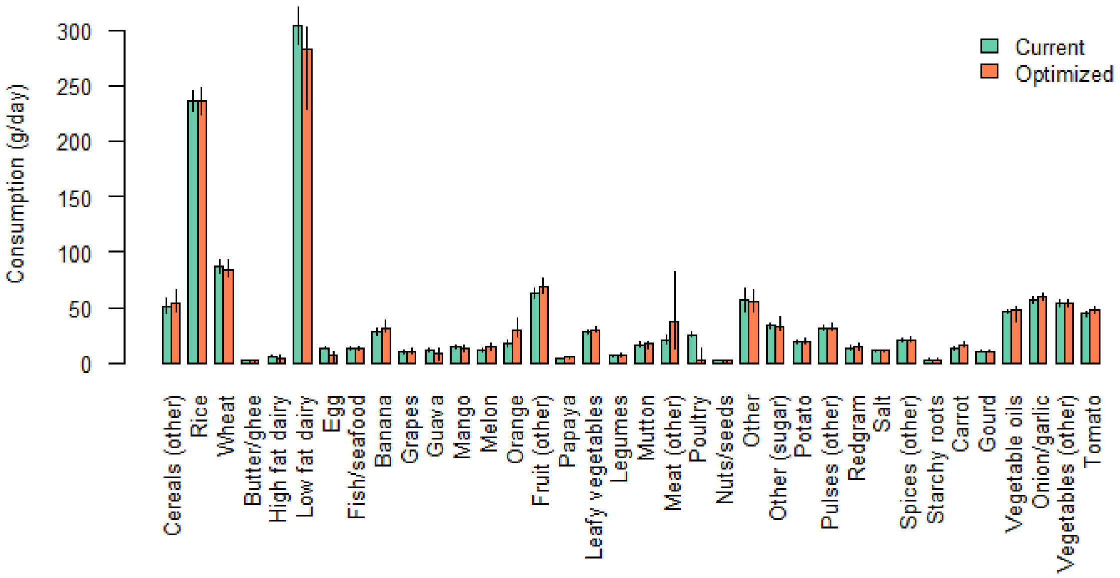

Figure S7. Average consumption of 36 food groups in g/day for *Rice and meat* pattern currently (green bars) and following optimization (orange bars) under 2025 scenario. Error bars = 95% confidence intervals.

Consumption in baseline and optimized dietary patterns: 2050 scenario

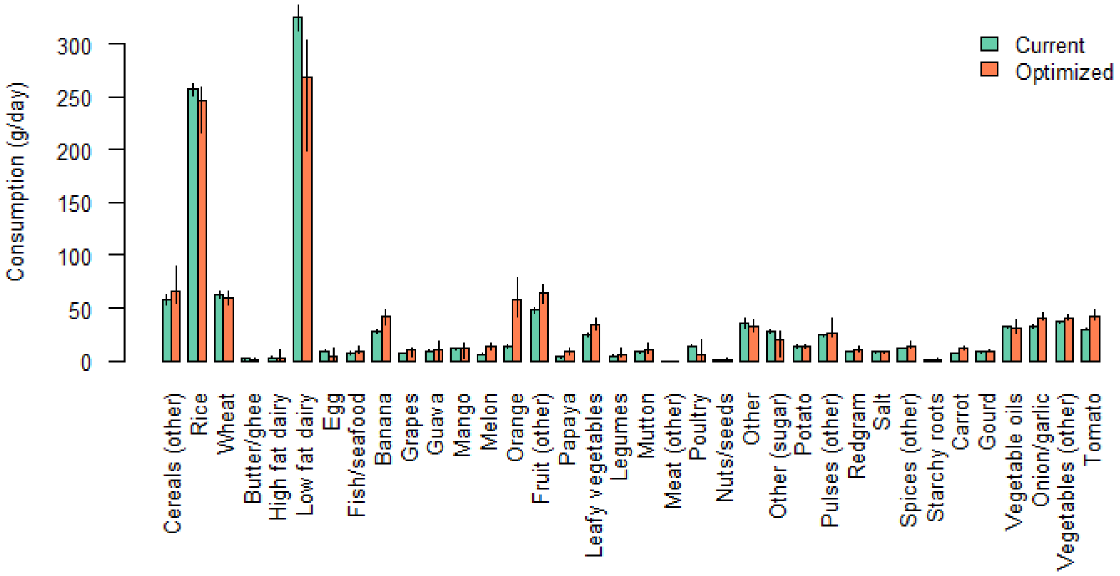

Figure S8. Average consumption of 36 food groups in g/day for *Rice and low diversity* pattern currently (green bars) and following optimization (orange bars) under 2050 scenario. Error bars = 95% confidence intervals.

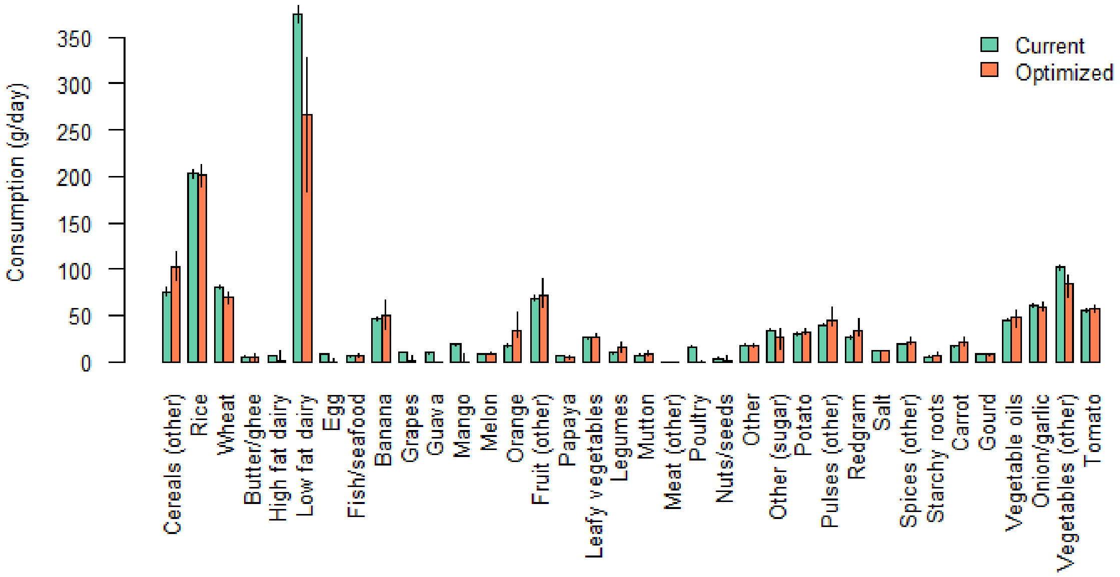

Figure S9. Average consumption of 36 food groups in g/day for *Rice and fruit* pattern currently (green bars) and following optimization (orange bars) under 2050 scenario. Error bars = 95% confidence intervals.

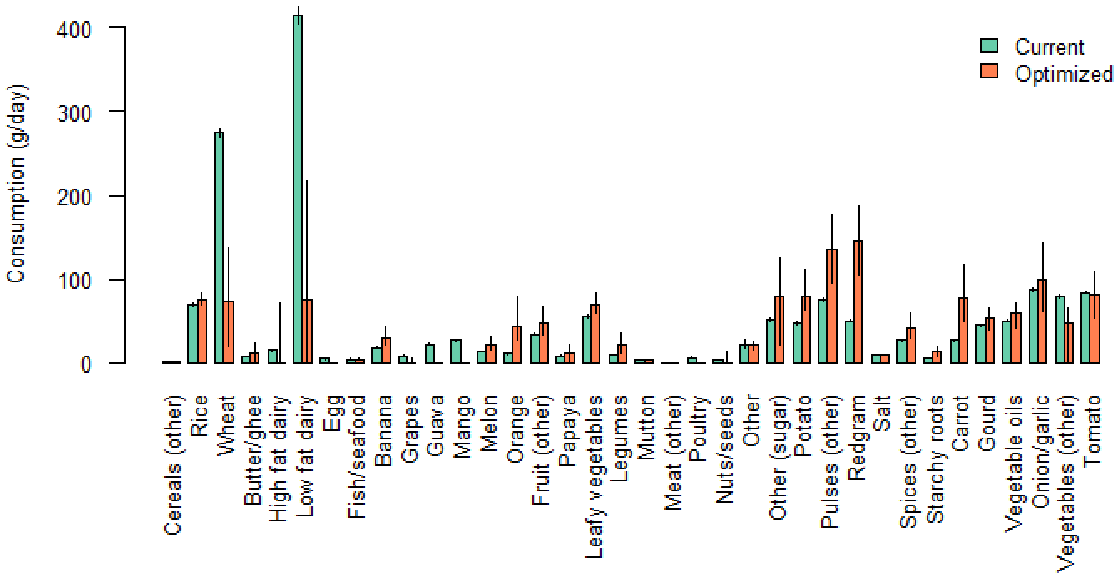

Figure S10. Average consumption of 36 food groups for *Wheat and pulses* pattern currently (green bars) and following optimization (orange bars) under 2050 scenario. Error bars = 95% confidence intervals.

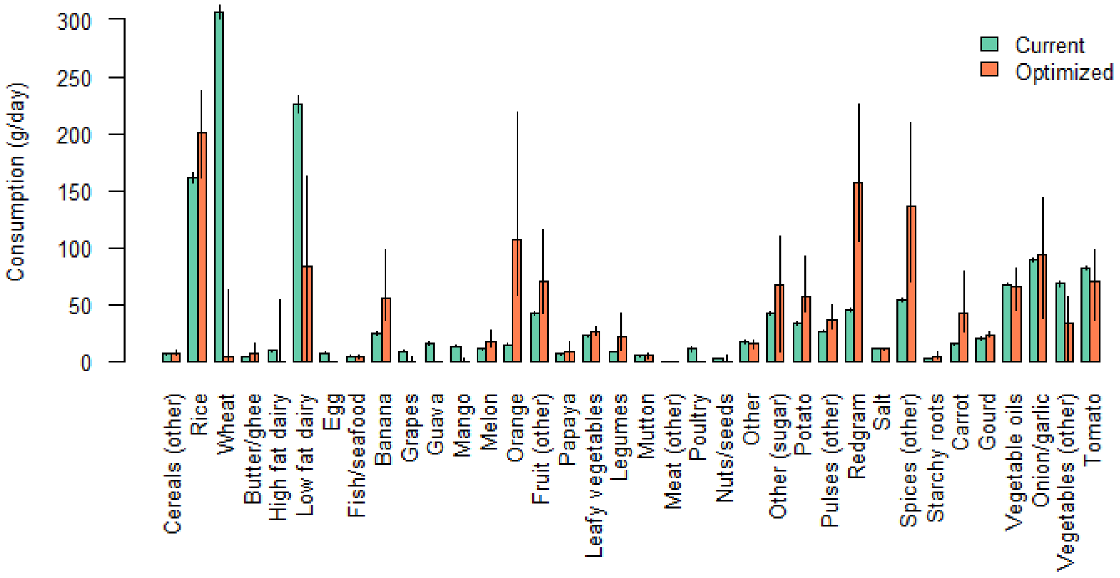

Figure S11. Average consumption of 36 food groups in g/day for *Wheat, rice and oils* pattern currently (green bars) and following optimization (orange bars) under 2050 scenario. Error bars = 95% confidence intervals.

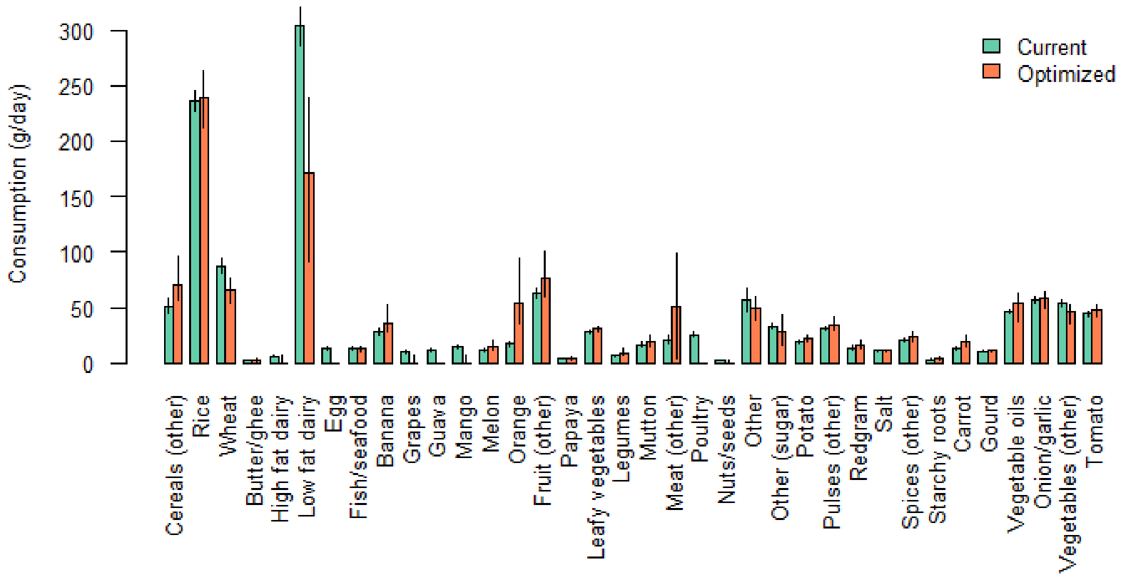

Figure S12. Average consumption of 36 food groups in g/day for *Rice and meat* pattern currently (green bars) and following optimization (orange bars) under 2050 scenario. Error bars = 95% confidence intervals.

Baseline levels of key food groups

Table S6. Mean consumption of key food groups in baseline Indian dietary patterns.

Dietary pattern	Baseline dietary consumption (g/day)			
	Fruits	Vegetables	Mutton and other red meat	Poultry
Rice and low diversity	128·1	140·3	8·9	14·3
Rice and fruit	185·6	269·6	7·4	16·2
Wheat and pulses	141·1	376·8	2·8	6·0
Wheat, rice and oils	136·7	297·4	4·8	10·7
Rice and meat	160·3	206·2	38·1	25·8

Changes in key food groups: 2025 scenario

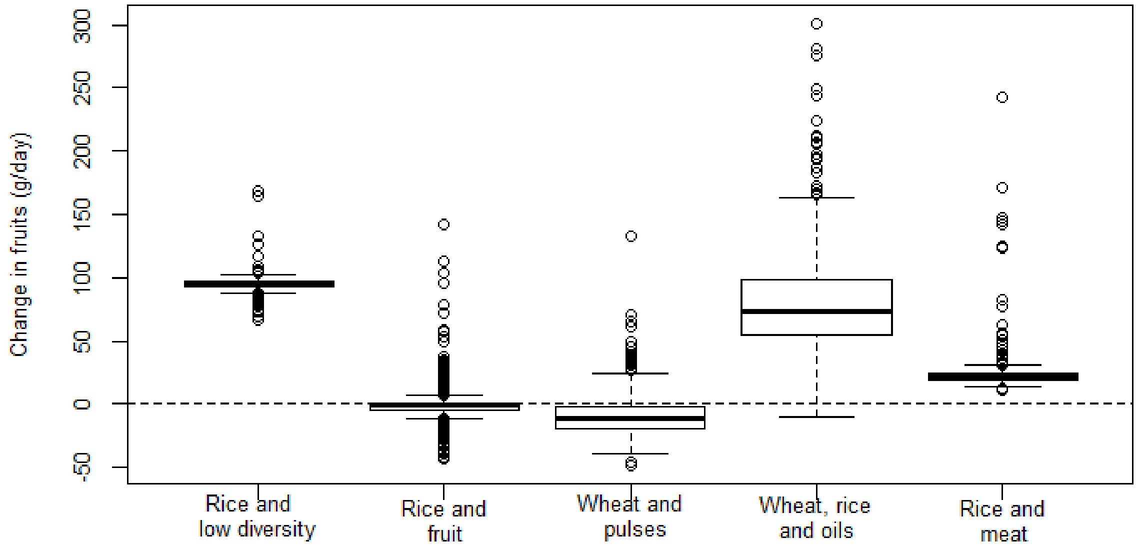

Figure S13. Changes in fruit consumption for each dietary pattern under 2025 scenario due to adoption of optimized Indian dietary. Thick lines = median; boxes = interquartile range; whiskers = limits of nominal range; open circles = outliers.

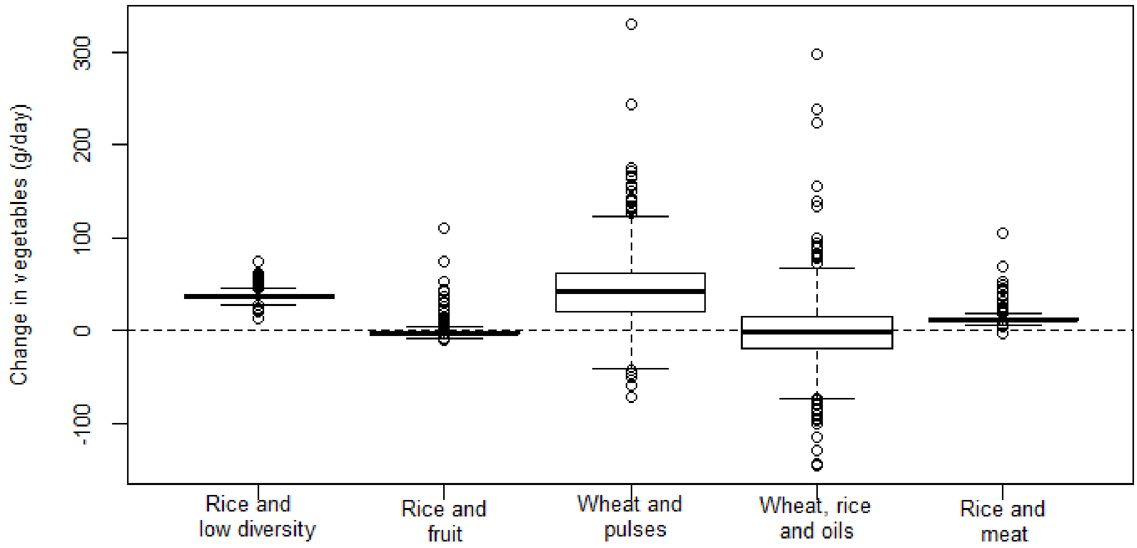

Figure S14. Changes in vegetable consumption for each dietary pattern under 2025 scenario due to adoption of optimized Indian dietary. Thick lines = median; boxes = interquartile range; whiskers = limits of nominal range; open circles = outliers.

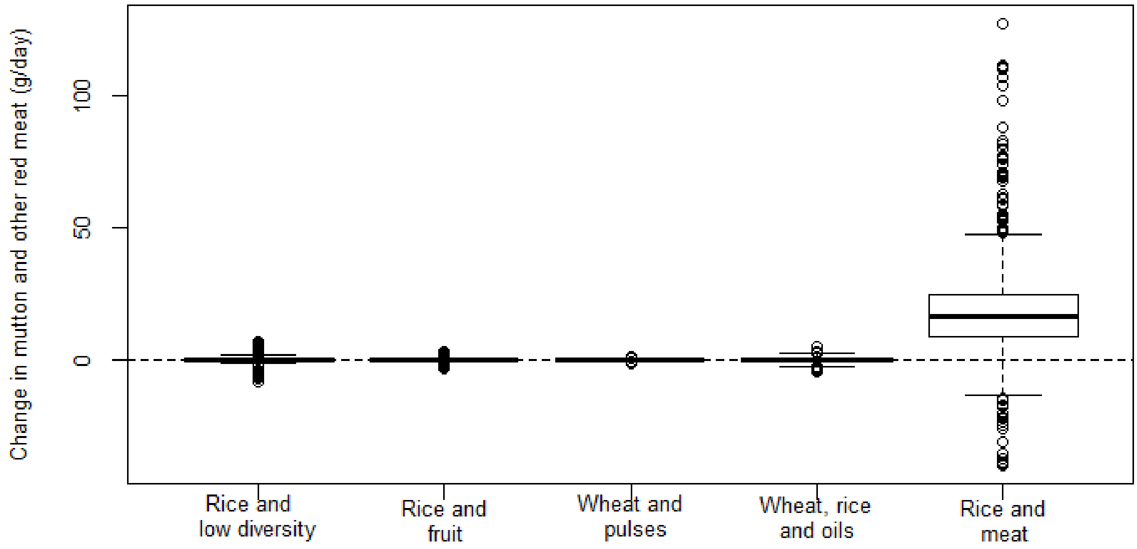

Figure S15. Changes in mutton and other red meat consumption for each dietary pattern under 2025 scenario due to adoption of optimized Indian dietary. Thick lines = median; boxes = interquartile range; whiskers = limits of nominal range; open circles = outliers.

Changes in key food groups: 2050 scenario

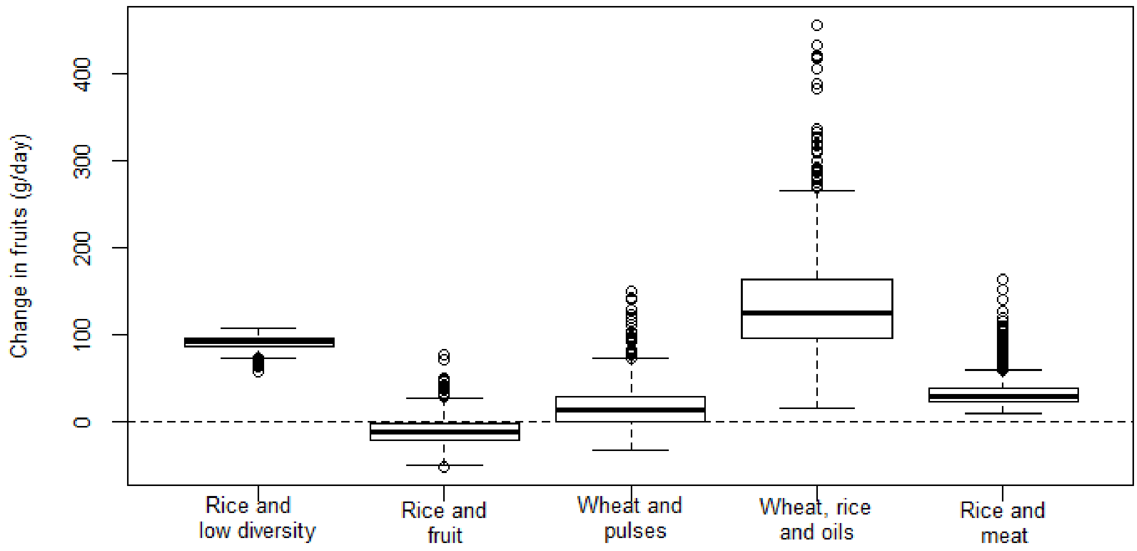

Figure S16. Changes in fruit consumption for each dietary pattern under 2050 scenario due to adoption of optimized Indian dietary. Thick lines = median; boxes = interquartile range; whiskers = limits of nominal range; open circles = outliers.

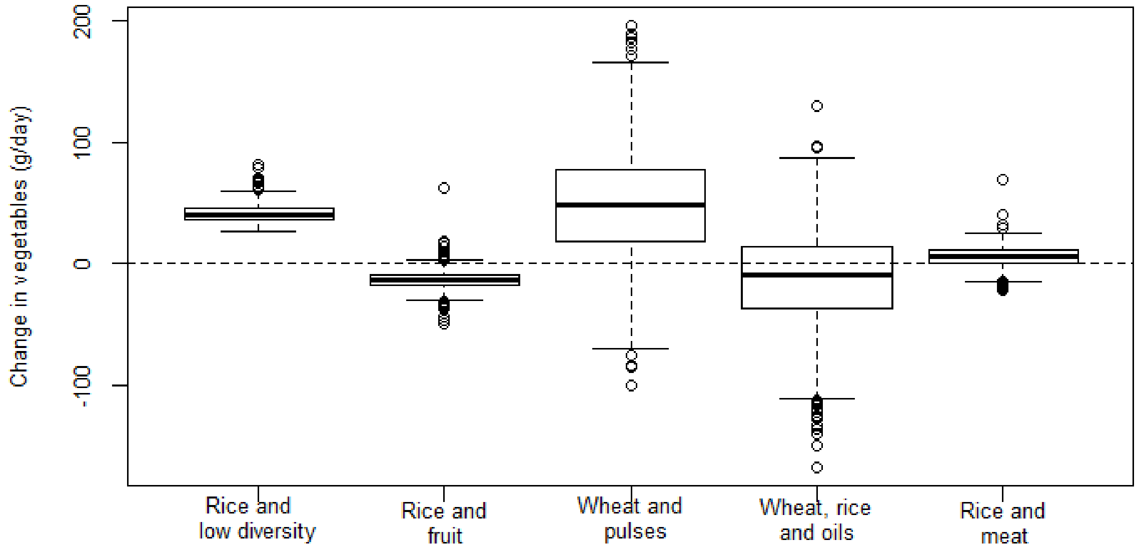

Figure S17. Changes in vegetable consumption for each dietary pattern under 2050 scenario due to adoption of optimized Indian dietary. Thick lines = median; boxes = interquartile range; whiskers = limits of nominal range; open circles = outliers.

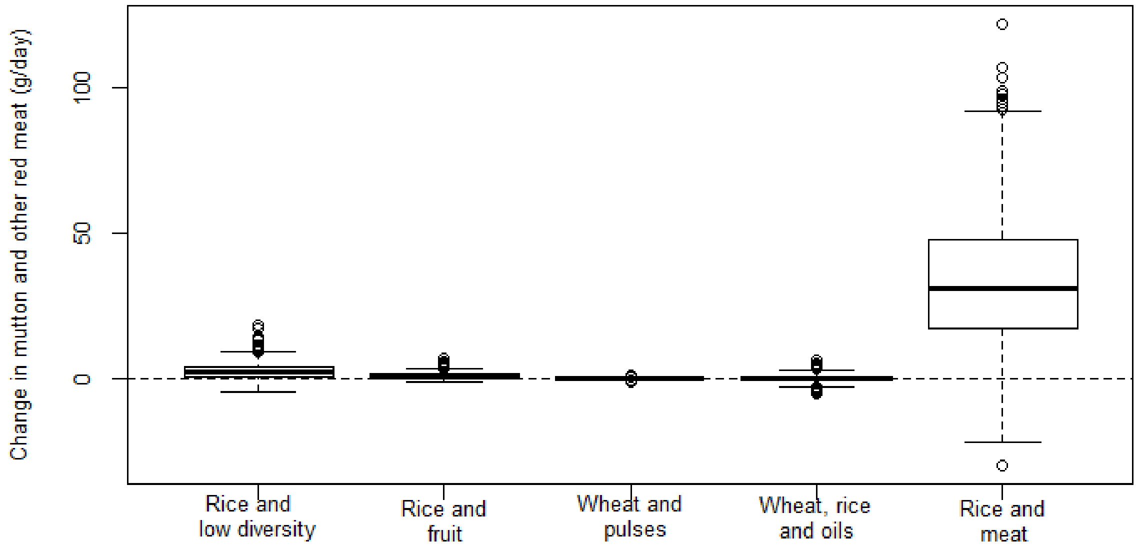

Figure S18. Changes in mutton and other red meat consumption for each dietary pattern under 2050 scenario due to adoption of optimized Indian dietary. Thick lines = median; boxes = interquartile range; whiskers = limits of nominal range; open circles = outliers.

Changes in blue water footprints: 2025 scenario

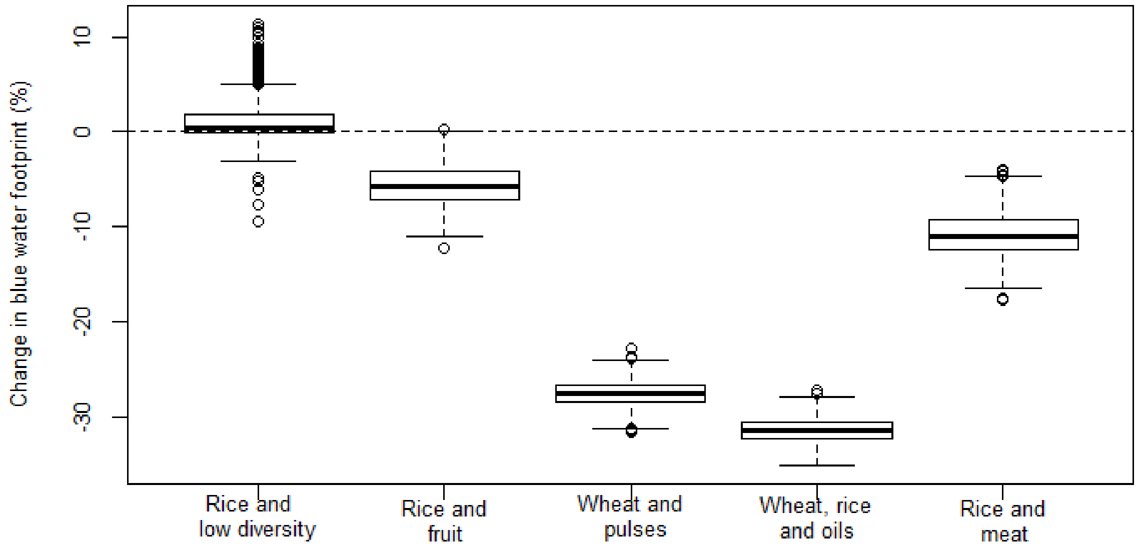

Figure S19. Percentage changes in blue water footprints for each dietary pattern under 2025 scenario due to adoption of optimized Indian dietary. Thick lines = median; boxes = interquartile range; whiskers = limits of nominal range; open circles = outliers.

Changes in blue water footprints: 2050 scenario

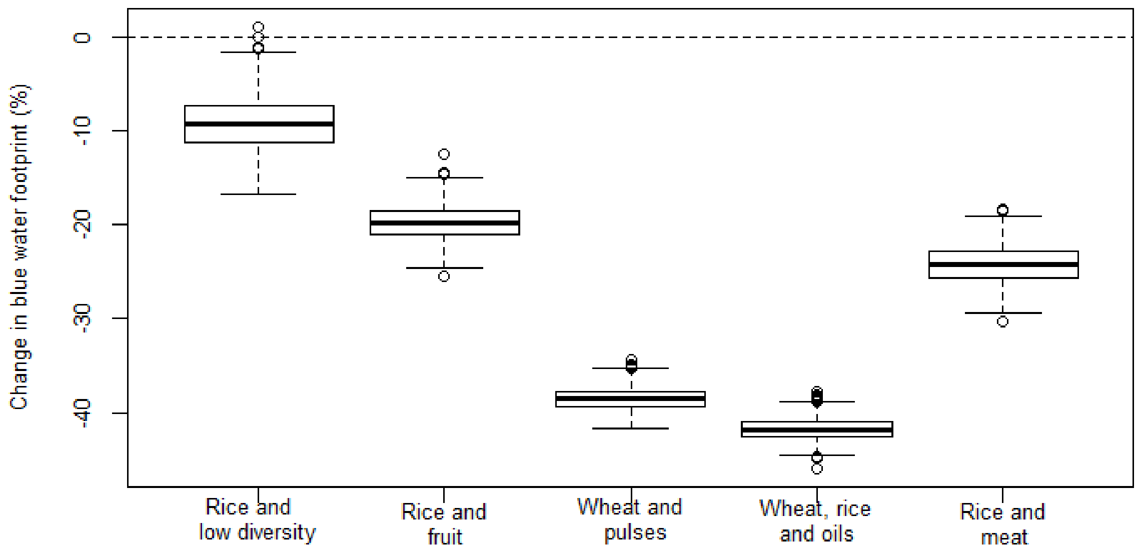

Figure S20. Percentage changes in blue water footprints for each dietary pattern under 2050 scenario due to adoption of optimized Indian dietary. Thick lines = median; boxes = interquartile range; whiskers = limits of nominal range; open circles = outliers.

Changes in GHG emissions: 2025 scenario

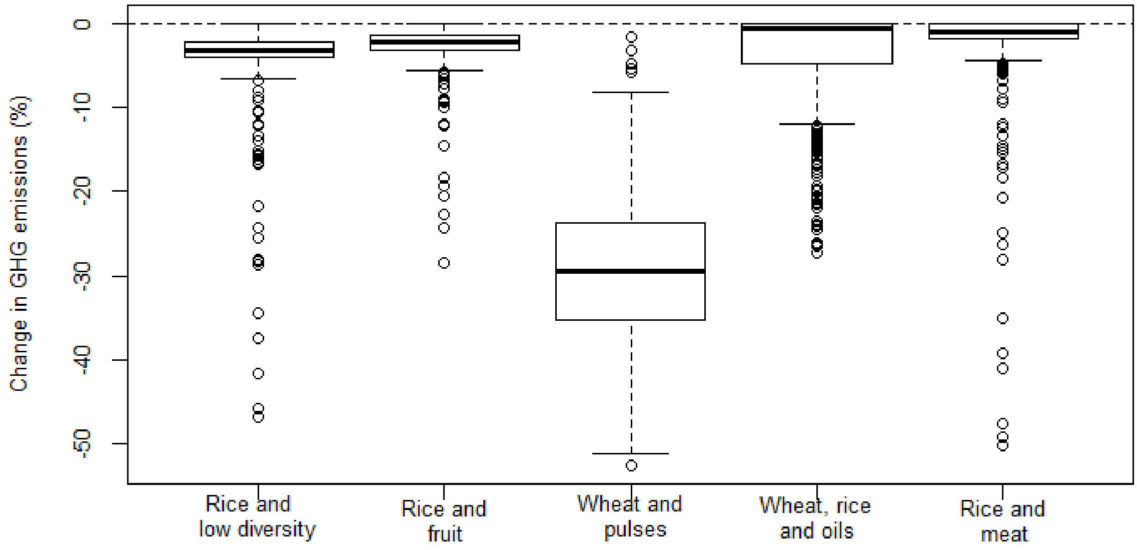

Figure S21. Percentage changes in GHG emissions for each dietary pattern under 2025 scenario due to adoption of optimized Indian dietary. Thick lines = median; boxes = interquartile range; whiskers = limits of nominal range; open circles = outliers.

Changes in GHG emissions: 2050 scenario

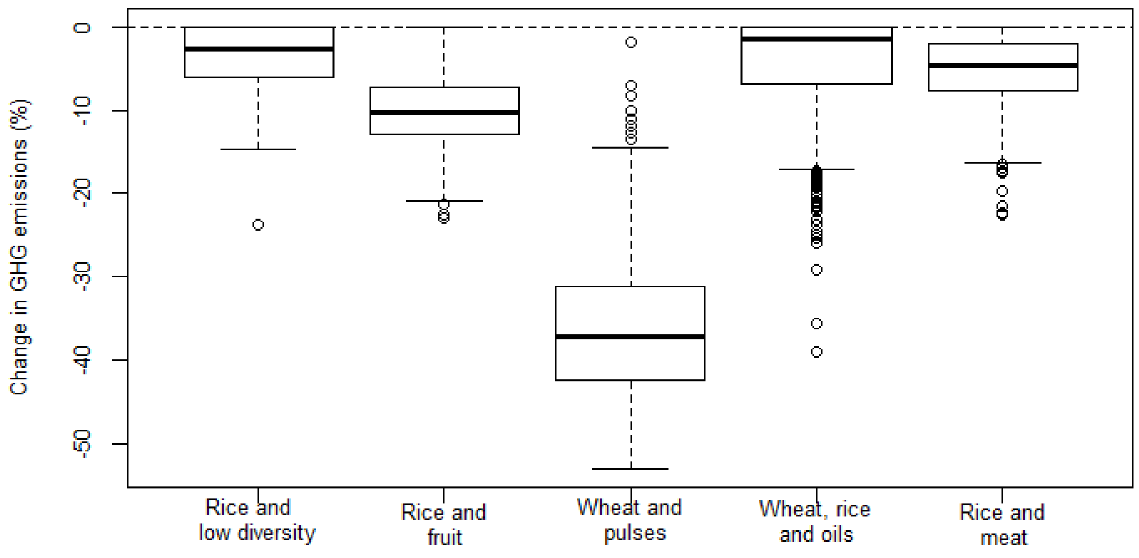

Figure S22. Percentage changes in GHG emissions for each dietary pattern under 2050 scenario due to adoption of optimized Indian dietary. Thick lines = median; boxes = interquartile range; whiskers = limits of nominal range; open circles = outliers.

Health impacts

Table S7. Modelled health impacts due to adoption of optimized Indian dietary patterns for 2025 and 2050 scenarios. Values are mean impacts and 95% confidence intervals based on Monte Carlo simulation.

Dietary pattern	Health impact (change in life years over 40 years per 100000 population)				
	CHD	Stroke	Cancers	Type 2 diabetes	Total
2025 scenario: Minimum 18·0% blue water footprint reduction					
Rice and low diversity	8950<br>(5886, 12590)	1122<br>(696, 1561)	3484<br>(2879, 4087)	-13<br>(-140, 34)	13543<br>(10386, 17413)
Rice and fruit	-245<br>(-1649, 2343)	-18<br>(-338, 280)	-19<br>(-524, 1065)	-7<br>(-57, 6)	-290<br>(-2524, 3711)
Wheat and pulses	3441<br>(-3511, 12507)	-125<br>(-462, 297)	871<br>(-1023, 3156)	-5<br>(-25, 8)	4182<br>(-4657, 15769)
Wheat, rice and oils	4332<br>(-2516, 12778)	926<br>(174, 1998)	2144<br>(171, 4629)	-3<br>(-86, 97)	7398<br>(-1257, 18854)
Rice and meat	2586<br>(1447, 4069)	-22<br>(-835, 440)	1053<br>(664, 1453)	-741<br>(-2677, 214)	2876<br>(-25, 5236)
2050 scenario: Minimum 30·3% blue water footprint reduction					
Rice and low diversity	9101<br>(5873, 12325)	1064<br>(623, 1525)	3453<br>(2872, 4061)	-106<br>(-411, 17)	13512<br>(10160, 16808)
Rice and fruit	-2101<br>(-4654, 798)	-168<br>(-624, 332)	-523<br>(-1303, 595)	-43<br>(-165, 11)	-2834<br>(-6261, 1507)
Wheat and pulses	5499<br>(-3074, 14717)	199<br>(-296, 955)	1671<br>(-661, 4137)	-7<br>(-28, 9)	7361<br>(-3477, 19356)
Wheat, rice and oils	6712<br>(-2017, 18119)	1565<br>(513, 3266)	3165<br>(848, 5850)	-6<br>(-115, 118)	11435<br>(846, 26263)
Rice and meat	2559<br>(1009, 5550)	-128<br>(-1219, 904)	1124<br>(632, 2574)	-1330<br>(-4216, 431)	2225<br>(-2337, 8154)

Total health impacts: 2025 scenario

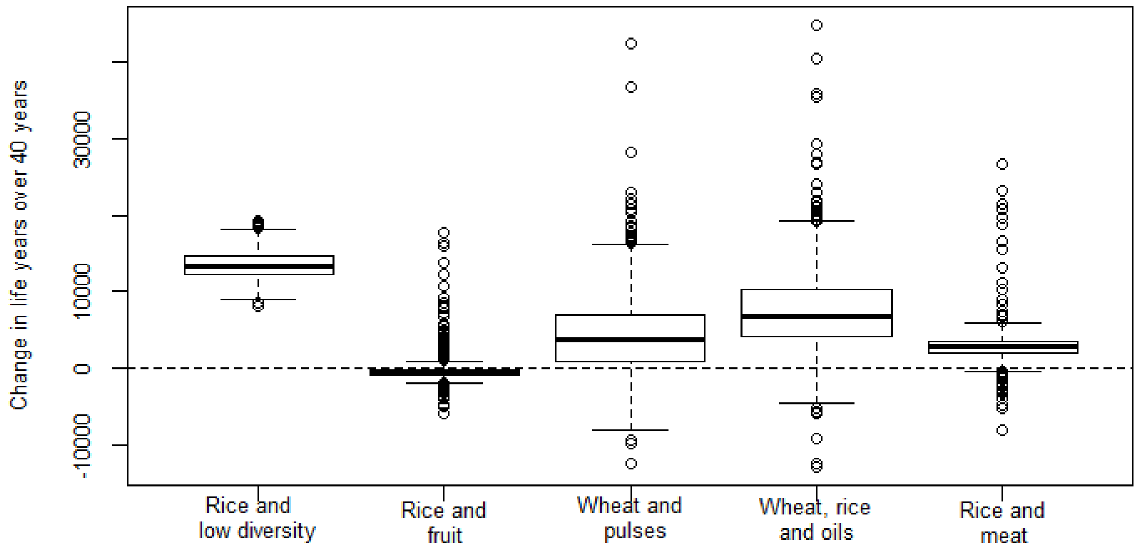

Figure S23. Total modelled health impact (changes in life years over 40 years per 100000 population) for each dietary pattern under 2025 scenario due to adoption of optimized Indian dietary. Positive values indicate health benefits. Thick lines = median; boxes = interquartile range; whiskers = limits of nominal range; open circles = outliers.

Total health impacts: 2050 scenario

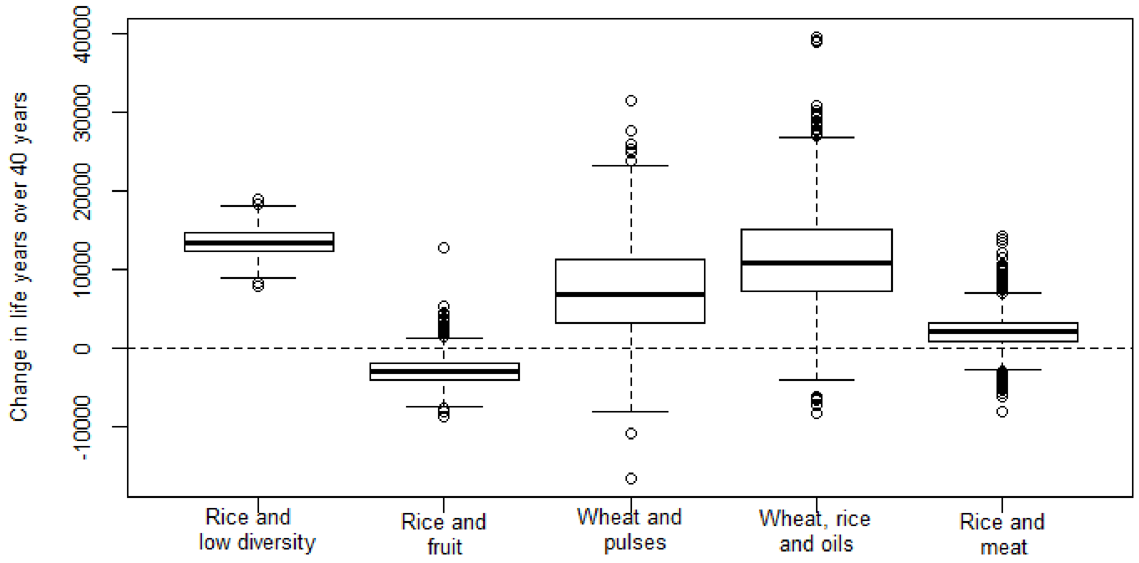

Figure S24. Total modelled health impact (changes in life years over 40 years per 100000 population) for each dietary pattern under 2050 scenario due to adoption of optimized Indian dietary. Positive values indicate health benefits. Thick lines = median; boxes = interquartile range; whiskers = limits of nominal range; open circles = outliers.

Micronutrient intake

Table S8. Mean baseline micronutrient intake in Indian dietary patterns.

Dietary pattern	Mean baseline intake			
	Calcium (mg)	Iron (mg)	Zinc (mg)	Vitamin A (µg)
Rice and low diversity	835	16·4	9·1	423
Rice and fruit	1043	20·5	10·6	679
Wheat and pulses	1122	23·5	10·3	875
Wheat, rice and oils	882	26·8	10·3	628
Rice and meat	952	21·3	11·1	854

Table S9. Mean changes in micronutrient intake due to adoption of optimized Indian dietary patterns for 2025 and 2050 scenarios.

Dietary pattern	Mean change in intake			
	Calcium (mg)	Iron (mg)	Zinc (mg)	Vitamin A (µg)
2025 scenario: Minimum 18·0% blue water footprint reduction				
Rice and low diversity	10·6	1·0	0·1	71·6
Rice and fruit	-17·0	0·2	-0·0	-7·5
Wheat and pulses	-216·9	1·3	0·1	242·3
Wheat, rice and oils	130·9	6·9	1·2	300·1
Rice and meat	-23·2	1·2	0·3	284·3
2050 scenario: Minimum 30·3% blue water footprint reduction				
Rice and low diversity	13·2	1·7	0.2	60·3
Rice and fruit	-86·7	0·8	-0·0	-46·4
Wheat and pulses	-231·1	2·0	0·4	378·8
Wheat, rice and oils	161·2	9·2	1·7	409·5
Rice and meat	133·0	2·1	0·5	414·8

References

1 Indian Ministry of Water Resources. *Strategic Plan for Ministry of Water Resources*. New Delhi: Indian Ministry of Water Resources, 2011.
2 Ebrahim S, Kinra S, Bowen L, et al. The effect of rural-to-urban migration on obesity and diabetes in India: a cross-sectional study. *PLOS Med* 2010; **7:** e1000268.
3 Bowen L, Bharathi AV, Kinra S, Destavola B, Ness A, Ebrahim S. Development and evaluation of a semi-quantitative food frequency questionnaire for use in urban and rural India. *Asia Pac J Clin Nutr* 2012; **21:** 355–60.
4 Gopalan C, Rama Sastri BV, Balasubramanian SC. *Nutritive Value of Indian Foods*. Hyderabad: National Institute of Nutrition, Indian Council of Medical Research, 1971.
5 USDA-ARS. *National Nutrient Database for Standard Reference, Release 28*. US Department of Agriculture, Agricultural Research Service. Available at: http://www.ars.usda.gov/nea/bhnrc/ndl
6 Fahey MT, Thane CW, Bramwell GD, Coward WA. Conditional Gaussian mixture modelling for dietary pattern analysis. *J R Stat Soc Ser A* 2007; **170:** 149–66.
7 Joy EJM, Green R, Agrawal S, et al. Dietary patterns and non-communicable disease risk in Indian adults: secondary analysis of Indian Migration Study data. *Public Health Nutr* 2017, Submitted.
8 Mekonnen MM, Hoekstra AY. The green, blue and grey water footprint of crops and derived crop products. *Hydrol Earth Syst Sci* 2011; **15:** 1577–600.
9 Mekonnen MM, Hoekstra AY. A global assessment of the water footprint of farm animal products. *Ecosystems* 2012; **15:** 401–15.
10 Wint GRW, Robinson TP. *Gridded Livestock of the World 2007*. Rome: Food and Agriculture Organization (FAO), 2007. http;//www.fao.org/docrep/010/a1259e/a1259e00.html
11 Steinfeld H, Gerber P, Wassenaar T, Castel V, Rosales M, de Haan C. *Livestock's Long Shadow: Environmental Issues and Options*. Rome: Food and Agriculture Organization (FAO), 2006. ftp://ftp.fao.org/docrep/fao/010/a0701e/A0701E.pdf
12 FAO. *Technical Conversion Factors for Agricultural Commodities*. Rome: Food and Agriculture Organization (FAO), 2003. http://www.fao.org/fileadmin/templates/ess/documents/methodology/tcf.pdf
13 Suresh AV. *Development of the Aquafeed Industry in India*. FAO Fisheries Technical Paper No. 497. Rome: Food and Agriculture Organization (FAO), 2007. http://www.fao.org/3/a-a1444e/a1444e08.pdf
14 Office of the Registrar General. *Census of India*. New Delhi, India: Ministry of Home Affairs, Government of India, 2011. Available at: http://www.censusindia.gov.in/2011census/hlo/pca/pca_pdf/PCA-CRC-0000.pdf
15 Harris F, Green RF, Joy EJM, Kayatz B, Haines A, Dangour AD. The water use of Indian diets and socio-demographic factors related to dietary blue water footprint. *Sci Total Environ* 2017, Accepted for publication.
16 Foster C, Green K, Bleda M, et al. *Environmental Impacts of Food Production and Consumption*. Manchester Business School, Manchester: Final Report to the Department for Environment, Food and Rural Affairs (DEFRA), 2006.
17 Audsley E, Brander M, Chatterton J, Murphy-Bokern D, Webster C, Williams A. *How Low Can We Go? An Assessment of Greenhouse Gas Emissions from the UK Food System and the Scope for Reduction by 2050*. WWF-UK, 2009.
18 FAO. *Global Food Losses and Food Waste: Extent, Causes and Prevention*. Rome: Food and Agriculture Organization (FAO), 2011. http://www.fao.org/docrep/014/mb060e/mb060e.pdf
19 Pathak H, Jain N, Bhatia A, Patel J, Aggarwal PK. Carbon footprints of Indian food items. *Agric Ecosyst Environ* 2010; **139:** 66–73.
20 Klenk I, Landquist B, Ruiz de Imana O. The product carbon footprint of EU beet sugar: summary of key findings. *Sugar Industry Journal* 2012; **137:** 1–17.
21 Williams AG, Audsley E, Sandars DL. *Determining the Environmental Burdens and Resource Use in the Production of Agricultural and Horticultural Commodities*. Report to the Department for Environment, Food and Rural Affairs (DEFRA), 2006.
22 Röös E, Karlsson H, Witthoft C, Sundberg C. Evaluating the sustainability of diets – combining environmental and nutritional aspects. *Environ Sci Policy* 2015; **57:** 157–66.
23 FAO. *FAOSTAT Food Balance Sheets*. Rome: Food and Agriculture Organization (FAO), 2016. http://faostat3.fao.org/
24 Hillier JG, Walter C, Malin D, Garcia-Suarez T, Mila-i-Canals L, Smith, P. A farm-focused calculator for emissions from crop and livestock production. *Environ Model Softw* 2011; **26;** 1070–8.
25 Vetter SH, Sapkota TB, Hillier J, et al. Greenhouse gas emissions from agricultural food production to supply Indian diets: Implications for climate change mitigation. *Agric Ecosyst Environ* 2017, Accepted for publication.

26 Green RF, Joy EJM, Harris F, et al. Greenhouse gas emissions and water footprints of typical dietary patterns in India: a modelling study. *J Clean Prod* 2017, Submitted.
27 WHO. *Diet, Nutrition, and the Prevention of Chronic Disease: Report of a Joint WHO/FAO Expert Consultation*. Geneva, Switzerland: World Health Organization, 2003.
28 National Sample Survey Office. *Nutritional Intake in India, 2004-05. NSS 61st Round*. New Delhi, India: Government of India, 2007.
29 Kumar P, Kumar A, Parappurathu S, Raju SS. Estimation of demand elasticity for food commodities in India. *Agricultural Economics Research Review* 2011; **24:** 1–4.
30 R Core Team. *R: A language and environment for statistical computing*. Contract No.: ISBN 3-900051-07-0 http://www.R-project.org/. Vienna, Austria: R Foundation for Statistical Computing.
31 Varadhan R. *Alabama: Constrained Nonlinear Optimization*. R Package Version 2011.9-1, 2012. http://CRAN.Rproject.org/package=Alabama
32 Green R, Milner J, Dangour AD, et al. The potential to reduce greenhouse gas emissions in the UK through healthy and realistic dietary change. *Clim Change* 2015; **129:** 253–65.
33 Milner J, Green R, Dangour AD, et al. Health effects of adopting low greenhouse gas emission diets in the UK. *BMJ Open* 2015; **5:** e007364.
34 Miller B, Hurley J. Life table methods for quantitative impact assessments in chronic mortality. *J Epidemiol Community Health* 2003; **57:** 200–6.
35 Office of the Registrar General. *Report of Causes of Death in India 2001-2003*. New Delhi, India: Ministry of Home Affairs, Government of India, 2009.
36 Office of the Registrar General. *Report of Medical Certification of Cause of Death 2012*. New Delhi, India: Ministry of Home Affairs, Government of India, 2015.
37 UNSD. *UNdata website*. New York, NY, USA: United Nations Statistics Division, 2015. http://data.un.org/Default.aspx
38 WHO. *Global Health Observatory website*. Geneva, Switzerland: World Health Organization, 2015. http://www.who.int/gho/en/
39 Vos T, Barber RM, Bell B, et al. Global, regional, and national incidence, prevalence, and years lived with disability for 301 acute and chronic diseases and injuries in 188 countries, 1990–2013: a systematic analysis for the Global Burden of Disease Study 2013. *Lancet* 2015; **386:** 743–800.
40 Dauchet L, Amouyel P, Dallongeville J. Fruit and vegetable consumption and risk of stroke: a meta-analysis of cohort studies. *Neurology* 2005; **65:** 1193–7.
41 Dauchet L, Amouyel P, Hercberg S, Dallongeville J. Fruit and vegetable consumption and risk of coronary heart disease: A meta-analysis of cohort studies. *J Nutr* 2006; **136:** 2588–93.
42 Marmot M, Atinmo T, Byers T, et al. Food, nutrition, physical activity, and the prevention of cancer: a global perspective. *World Cancer Res Fund/Am Inst Cancer* 2007; **46:** 312–14.
43 Pan A, Sun Q, Bernstein AM, et al. Red meat consumption and risk of type 2 diabetes: 3 cohorts of US adults and an updated meta-analysis. *Am J Clin Nutr* 2011; **94:** 1088–96.
44 Micha R, Wallace SK, Mozaffarian D. Red and processed meat consumption and risk of incident coronary heart disease, stroke and diabetes mellitus - a systematic review. *Circulation* 2010; **121:** 2271-83.
45 Capewell S, O'Flaherty M. Can dietary changes rapidly decrease cardiovascular mortality rates? *Eur Heart J* 2011; **32:** 1187–9.
46 Franco M, Ordunez P, Caballero B, et al. Impact of energy intake, physical activity, and population-wide weight loss on cardiovascular disease and diabetes mortality in Cuba, 1980-2005. *Am J Epidemiol* 2007; **166:** 1377–80.
47 Harashima E, Nakagawa Y, Urata G, Tsuji T, Shirataka M, Matsumura Y. Time-lag estimate between dietary intake and breast cancer mortality in Japan. *Asia Pac J Clin Nutr* 2007; **16:** 193–8.
48 Tsuji K, Harashima E, Nakagawa Y, Urata G, Shirataka M. Time-lag effects of dietary fiber and fat intake ratio on Japanese colon cancer mortality. *Biomed Environ Sci* 1996; **9:** 223–8.
